# Detection and Inhalation Hazard Assessment of Wear Particles Arising From Abrasion of Market‐relevant Graphene Reinforced Composites

**DOI:** 10.1002/smll.76506

**Published:** 2026-10-30

**Authors:** Govind Gupta, Jimmy Vernaz, Antje Vennemann, Maike Stange, Asmus Meyer‐Plath, Servane Contal, Sarah Zehnder, Ziting Wang, Aline Chary, Stephanie Eitner, Philipp Mueller, Vera M. Kissling, Samuel Constant, Lucie Chupin, Daniel Breitenstein, Birgit Hagenhoff, Tommaso Serchi, Bernadett Boda, Martin Wiemann, Peter Wick, Tina Buerki‐Thurnherr

**Affiliations:** ^1^ Nanomaterials in Health Laboratory Swiss Federal Laboratories For Materials Science and Technology (Empa) St. Gallen Switzerland; ^2^ Epithelix Sàrl Geneva Switzerland; ^3^ IBE R&D Institute for Lung Health GmbH Münster Germany; ^4^ Materials and Particulate Hazardous Substances Federal Institute For Occupational Safety and Health (BAuA) Berlin Germany; ^5^ Luxembourg Institute of Science and Technology (LIST) Belvaux Luxembourg; ^6^ BASF SE, Solid‐State Microstructures Ludwigshafen am Rhein Germany; ^7^ Carbon Waters Pessac France; ^8^ Tascon GmbH Münster Germany

**Keywords:** composite material, epoxy, graphene, in vitro toxicology, inhalation exposure, lung toxicity, materials science, nanocomposite

## Abstract

Commercial products incorporating graphene‐based two‐dimensional (2D) nanosheets are increasingly developed to enhance mechanical, thermal, and electrical performance. Potential release of inhalable particles during manufacturing or end‐of‐life processing, however, raises concerns regarding respiratory health. This study explored the pulmonary cytotoxicity and immunomodulatory activity of airborne particles generated by mechanical abrasion of industrially relevant few‐layer graphene (FLG) reinforced epoxy composites. A multimodal analytical strategy combining high‐resolution transmission electron microscopy, Raman microscopy, and time‐of‐flight secondary ion mass spectrometry enabled reliable detection and spatial mapping of FLG in pristine and abraded composites. Abrasion predominantly produced composite fragments in which FLG remained embedded, with no indication of protruding or freely released FLG nanosheets. Across advanced bronchial and alveolar epithelial air–liquid interface models, neither abraded epoxy nor FLG–epoxy particles induced cytotoxic or pro‐inflammatory responses. A moderate reduction of mucociliary clearance was observed only at high exposure doses of abraded epoxy particles, without amplification by FLG reinforcement. As‐produced FLG triggered strong TNF‐α secretion in alveolar macrophages, whereas abraded FLG–epoxy particles elicited minimal responses. Overall, the findings indicate limited acute respiratory toxicity associated with inhalation of fragments released from FLG‐based epoxy composites under production and end‐of‐life scenarios.

## Graphical abstract

Abrasion of few‐layer graphene (FLG)–epoxy composites generated respirable particles in which FLG remained fully embedded, as confirmed by correlative HR‑TEM, Raman, and ToF‑SIMS analyses. Advanced lung cell models revealed no cytotoxic or inflammatory responses, and only high‑dose epoxy particles reduced mucociliary clearance. Findings indicate minimal acute respiratory hazard from FLG‑epoxy fragments.

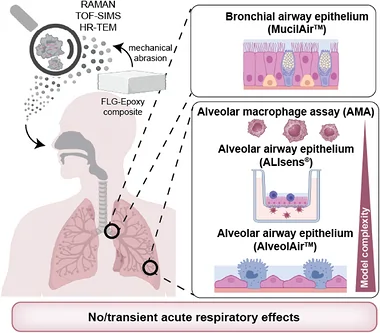

## 1 Introduction

The industrial and consumer products containing two‐dimensional (2D) graphene nanosheets are increasing in the market, as their incorporation into composite materials can enhance the properties of the product, specifically electro‐ and thermal conductivity, rigidity, lightness, and durability [1, 2, 3, 4]. Because graphene imparts performance‐enhancing properties to products, it is considered an advanced material according to the OECD definition (*“Advanced Materials: Working Description,”* ENV/CBC/MONO(2022)29) and related literature [5]. It is conceivable that small fragments released from graphene‐reinforced composites during wear processes (e.g. mechanical abrasion) throughout production, use or end‐of‐life stages of a product may become airborne, be inhaled, and exhibit biological behaviors distinct from those of the as‐produced or neat material. Therefore, transformations of graphene occurring during product manufacture, use and disposal should be considered prior to investigating their potential pulmonary toxicity in humans. However, the detection of graphene and its spatial distribution analysis in multicomponent product matrices or biological systems is challenging as it requires finding the carbon graphene material present at relatively low quantities (1%–5%) in an organic, mainly carbonaceous matrix (e.g. Epoxy) [6].

In previous studies, Raman spectroscopy has been successfully applied for the detection of as‐produced graphene and its composites based on their unique spectral signature (D, G, and 2D bands) [7, 8, 9, 10]. Shojaee et al. (2013) [11] showed dispersion and distribution of graphene platelets reinforced in epoxy polymer composites (mimicking a market product) using Raman‐confocal mapping. Furthermore, Chortarea et al. (2022) [12] identified the presence of reduced graphene oxide (rGO) in abraded composites of polyamide 6 (PA6) using Raman spectroscopy. Raman‐confocal mapping has also been used for the detection of graphene and its derivatives in biological systems. In this context, Syama et al. (2017) [13] showed organ distribution, epithelial barrier translocation (e.g., blood‐brain barrier), and clearance of graphene oxide in mice by mapping of tissues from the brain, liver, kidney, spleen, and bone marrow using Raman spectroscopy coupled with confocal microscopy (CRM). A similar analytical approach was also applied by others for the successful detection of graphene uptake and biodegradation in the lung [14, 15] and immune cells [16, 17, 18].

The cytotoxicity and immunomodulatory effects of as‐produced graphene‐based 2D materials have been extensively studied (as reviewed by Lin et al. (2024) [6]; Fadeel et al. [19]; Martin et al. 2019 [20]). However, there are limited conclusive studies on the safety of graphene‐reinforced polymer composites fragments released during the life cycle of an industrial product [21]. For instance, Chortarea et al. (2022) [12] demonstrated a limited and transient inflammatory response with no cytotoxicity in lung and immune cells in a comprehensive study involving in vivo and in vitro screening of abraded rGO‐PA6 composites. Netkueakul et al. (2019) [22] also demonstrated that the epoxy polymer composites of GO, rGO, and graphene nanoplatelets (GNP) released from abrasion did not trigger any cytotoxicity or oxidative stress in macrophage‐like cells (differentiated THP1 cell line). Abraded epoxy composites incorporating other carbon‐based materials, such as multiwalled carbon nanotubes (MWCNTs), have also shown no acute cytotoxicity in lung cells [23, 24].

Taking into account the limited understanding of the transformation and safety of graphene composites during production and end‐of‐life stages, this study aimed to explore and develop a multimodal analytical strategy for improved detection of graphene in complex samples, combined with an evaluation of acute biological responses. To this end, we combined different analytical imaging techniques (e.g., Raman spectroscopy, transmission electron microscopy (TEM) and time‐of‐flight secondary ion mass spectrometry (ToF‐SIMS)) to detect and visualize the spatial distribution of few‐layer graphene (FLG) within epoxy matrices. Since inhalation represents one of the major routes of exposure in occupational settings, we investigated potential inhalation hazards of associated with particles released by mechanical abrasion of FLG‐reinforced epoxy composites (Scheme 1). Cytotoxic, subtoxic (mucociliary clearance, barrier integrity, sensitization), and immunomodulatory effects were examined using the rat alveolar macrophage assay (AMA; NR8383 cells grown under submerged conditions) and advanced multicellular air–liquid interface bronchial (MucilAir) and alveolar (ALIsens and AlveolAir) in vitro models. Abraded FLG–epoxy particles were evaluated relative to abraded epoxy and as‐produced FLG under single or repeated exposures across a broad concentration range (0–100 µg cm^2^), selected to encompass both occupationally relevant low‐dose levels of graphene‐related materials [25] and higher concentrations to enable robust dose–response assessment and identification of potential hazard thresholds.

**SCHEME 1:**
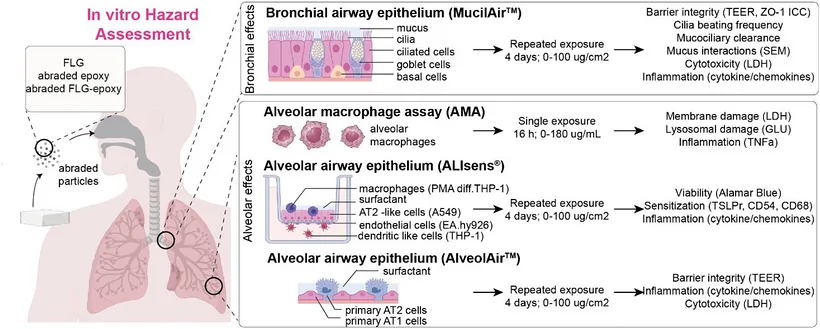
Overview of the study design investigating inhalation hazards of abraded FLG‐reinforced epoxy composites using macrophage and advanced bronchial/alveolar air–liquid interface in vitro models.

Our results demonstrated spatial distribution of FLG within the epoxy composite matrix using CRM and ToF‐SIMS. Alveolar macrophages largely ingested the materials and exhibited material‐specific variations in bioresponse. Notably, FLG was hardly cytotoxic but induced a pronounced secretion of the pro‐inflammatory tumor necrosis factor‐alpha (TNF‐α). In contrast, abraded particles of FLG‐epoxy composites showed a limited TNF‐α response but induced dose‐dependent cytotoxicity in alveolar macrophages. In the bronchial and alveolar epithelial cells cultures, neither FLG alone nor abraded FLG or epoxy composites decreased cell viability or compromised barrier integrity. However, a moderate reduction of mucociliary clearance was observed through real‐time in situ measurements and by scanning electron microscopy (SEM) images of cultures exposed to abraded epoxy composites. Overall, the in vitro data indicate that incorporating few‐layer graphene nanosheets into the epoxy matrix does not enhance the hazardous potential of the tested abraded polymer particles. Under the investigated exposure conditions, particles released from the FLG–epoxy composite induced limited acute adverse responses in the applied lung cell models.

## 2 Results and Discussion

### 2.1 Physico‐Chemical Characteristics of As‐Produced FLG Nanosheets

As shown in Figure 1, the as‐produced FLG nanosheets were characterized using high‐resolution (HR) TEM, Raman spectroscopy, and dynamic light scattering (DLS). HR‐TEM indicates a sheet‐like structure with an average lateral diameter of 631 ± 368 nm (± SD) (Figure 1a). Moreover, 10–12 layers were observed, with a step height between layers of approximately 0.32 nm (Figure 1b), which is close to that of bulk graphite (0.335 nm) [26]. To achieve a stable suspension of FLG nanosheets (1 mg mL^−1^) for biological experiments, 0.1% bovine serum albumin (BSA) was used as a stabilizer, as was also successfully applied earlier in preparing dispersions of 2D materials [12]. The presence of BSA in the FLG suspension was confirmed by UV–vis spectroscopy, showing signature spectra of BSA at 200–220 nm and 276 nm (Figure 1c). In addition, the Raman spectra typically indicated signatures of graphene flakes with a specific D band at 1309 cm^−1^; as well as G, D' and 2D bands at 1585, 1615, and 2613 cm^−1^, respectively (Figure 1d). The 2D band was well fitted by a single Lorentzian with a full width at half maximum (FWHM) of 45 cm^−1^, which is characteristic of a dispersion of few‐layer to multilayer graphene [27]. The ID/IG ratio was 2.4 and the FWHM of the D and G bands were 32 and 27 cm^−1^, respectively. These values correspond to a distance between defects (LD) of approximately 3–5 nm for 1.58 eV laser and a percentage of defects of approximately 0.1% to 0.4% (Figure 1d), as also shown elsewhere [28]. The average hydrodynamic size of the FLG suspension was 690 ± 88 nm (± SD), as determined using a Zetasizer DLS (Figure 1e).

**FIGURE 1:**
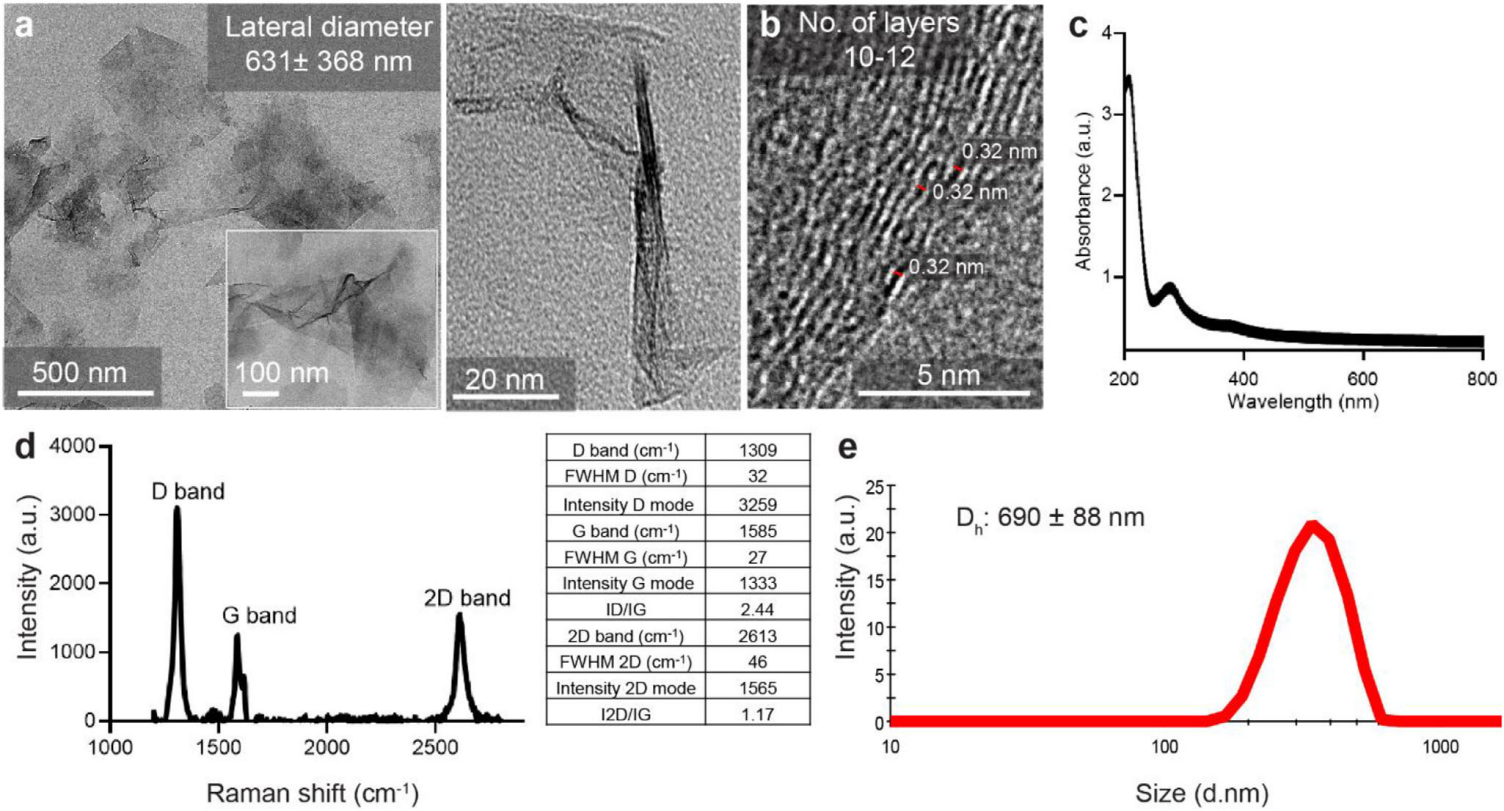
Characterization of the FLG suspension in 0.1% BSA. (a,b) HR‐TEM, (c) UV–vis spectrum showing absorbance, (d) Raman spectrum, (e) average hydrodynamic size measured using dynamic light scattering (DLS). A.u.: arbitrary units.

### 2.2 Detection and Physical Transformation of FLG in an Epoxy Product Matrix Under Simulated Life Cycle Conditions

The FLG mixed into epoxy can undergo multiple phases of physical and chemical transformations from production to the end of life. Therefore, although the detection and characterization of FLG in such complex matrices is challenging, it is crucial for understanding the behavior of FLG during the product life cycle. To investigate this, we first fabricated solid plates (23 × 21 × 0.4 cm) composed of epoxy (product matrix) reinforced with 1% (w/w) FLG, representing a market‐relevant product class, as FLG is increasingly used in thermally conductive coatings and adhesive formulations (Scheme S1). We also prepared epoxy plates without FLG to assess potential interference from the product matrix in the analytical methods. Epoxy was chosen as a product matrix since it is one of the most widely used materials due to its thermostatic and adhesive properties [29, 30]. Importantly, the laboratory‐based composite system enabled full control over the production process and composition, including additives. To mimic an end‐of‐life scenario, we performed mechanical abrasion of the fabricated plates by Taber abrasion, and particles released during abrasion were collected and used in further analysis (Scheme S1).

To detect FLG and its physical transformation in epoxy plates (mimicking a market product) and in abraded particles, we used CRM, ToF‐SIMS and HR‐TEM (Figures 2, 3, 4). The Raman spectroscopic analysis enabled the successful detection of FLG within the epoxy composite matrix (Figure 2). 3D depth profiling of FLG reinforced epoxy plates localized FLG within the product matrix and further revealed its spatial distribution indicating agglomerates embedded in the epoxy (Figure 2a and Video S1). Moreover, Raman spectra recorded from the abraded FLG‐epoxy composites particles showed the presence of typical FLG signatures with D (1309 cm^−1^), G (1585 cm^−1^) and 2D (2613 cm^−1^) bands at respective positions, with no interference from epoxy matrix spectra (Figure 2b). These results suggest that Raman spectroscopy can be effectively applied to detect FLG in complex product matrices, as well as after the degradation of commercial products. Although no changes were observed in the characteristic Raman spectra of FLG within the epoxy matrix, particle agglomeration was evident in the composite, indicating a physical transformation of FLG (Figure 2a). This agglomeration within the epoxy matrix may have implications at the end‐of‐life stage, as abraded particles (FLG–epoxy) could contain locally elevated FLG concentrations. Such enriched particles may lead to increased FLG exposure if taken up by environmental organisms or inhaled into the human respiratory system. Previous studies have applied Raman spectroscopy for the detection and characterization of graphene (incl. few to multilayer graphene) in commercial polymer composites, such as solar cells [31], optical sensors [32] and potassium ion barriers [33]. It has also been applied to identify graphene in degraded polymer matrices [12, 22, 34]. Chortarea et al. (2022) [12] detected reduced graphene oxide (rGO) in abraded polyamide 6 composites, while Netkueakul et al. (2020) [22] identified rGO in mechanically abraded epoxy composites. Additionally, Goodwin Jr et al. (2020) [34] applied Raman spectroscopy to study the degradation profile of FLG (3%) reinforced thermoplastic polyurethane (TPU) composites under ultraviolet (UV) light irradiation and found that FLG helped to protect the polymer from degradation under both dry and humid conditions.

**FIGURE 2:**
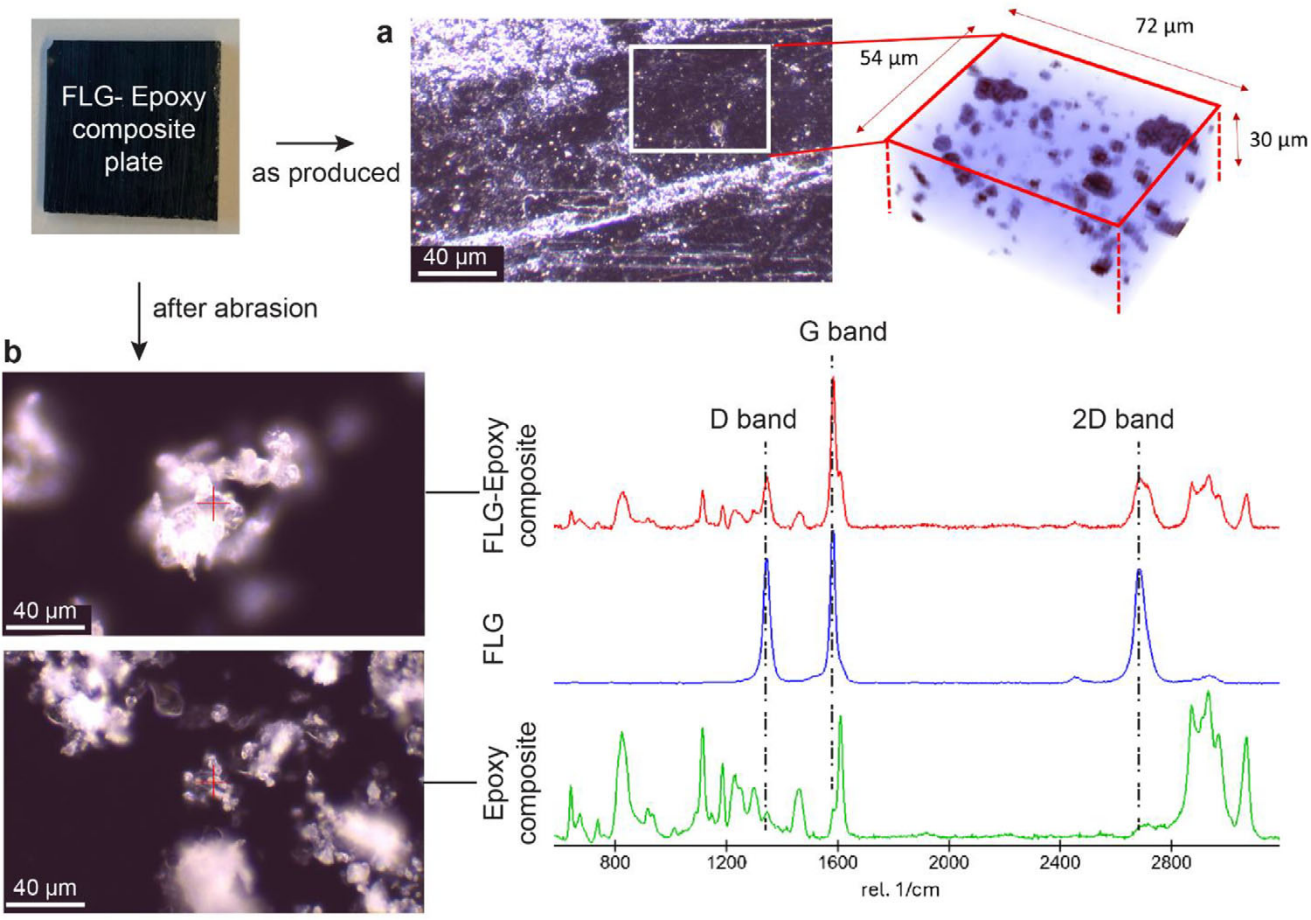
Detection of FLG in an epoxy polymer composite using confocal Raman microscopy. (a) Spatial distribution of FLG (1%) in epoxy composite plate reconstructed from a large area 3D‐depth profiling; the analyzed region is indicated in the darkfield image (left). The reconstructed 3D image is based on recorded FLG signature Raman spectra and shows the distribution of FLG (grey) in the epoxy matrix (blue). An additional 3D animation (Video S1) provides further insights into the FLG distribution in the product matrix. (b) Representative Raman spectra of abraded FLG‐epoxy and epoxy composite particles; selected regions are marked by red crosses in dark field images (left). Comparison with the as‐produced FLG confirmed successful detection of FLG in the epoxy matrix, with no interference from the background product matrix.

**FIGURE 3:**
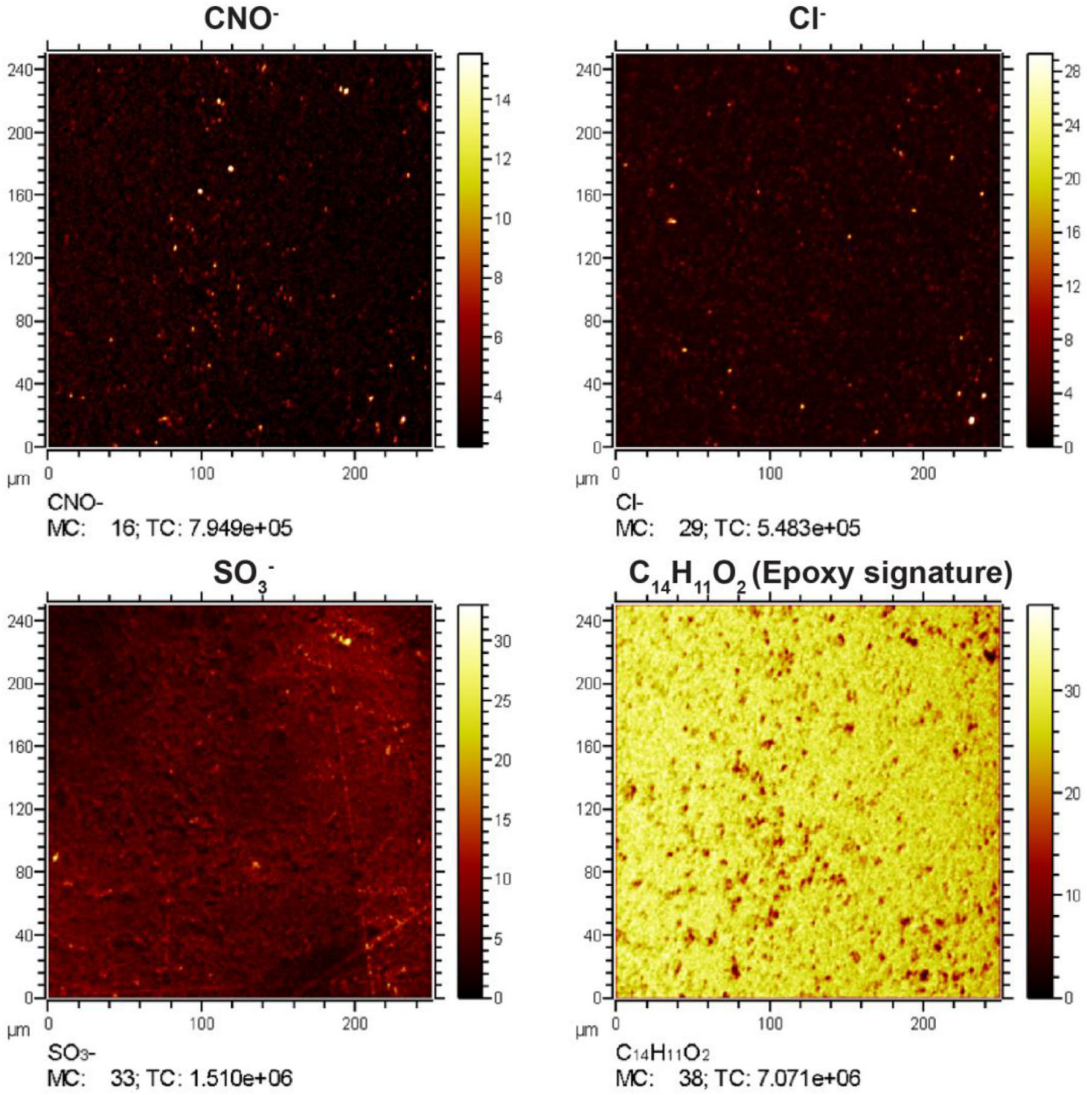
ToF‐SIMS 3D depth‐profiling of the epoxy plates containing 1% FLG did not allow a direct detection of graphene‐specific fragments. However, dotted structures showing elevated CNO^−^ and Cl^−^ intensities spatially coincide with the features observed in Raman maps (compare Figure 2), suggesting that these ToF‐SIMS signals may be associated with the presence or distribution of FLG within the epoxy matrix.

**FIGURE 4:**
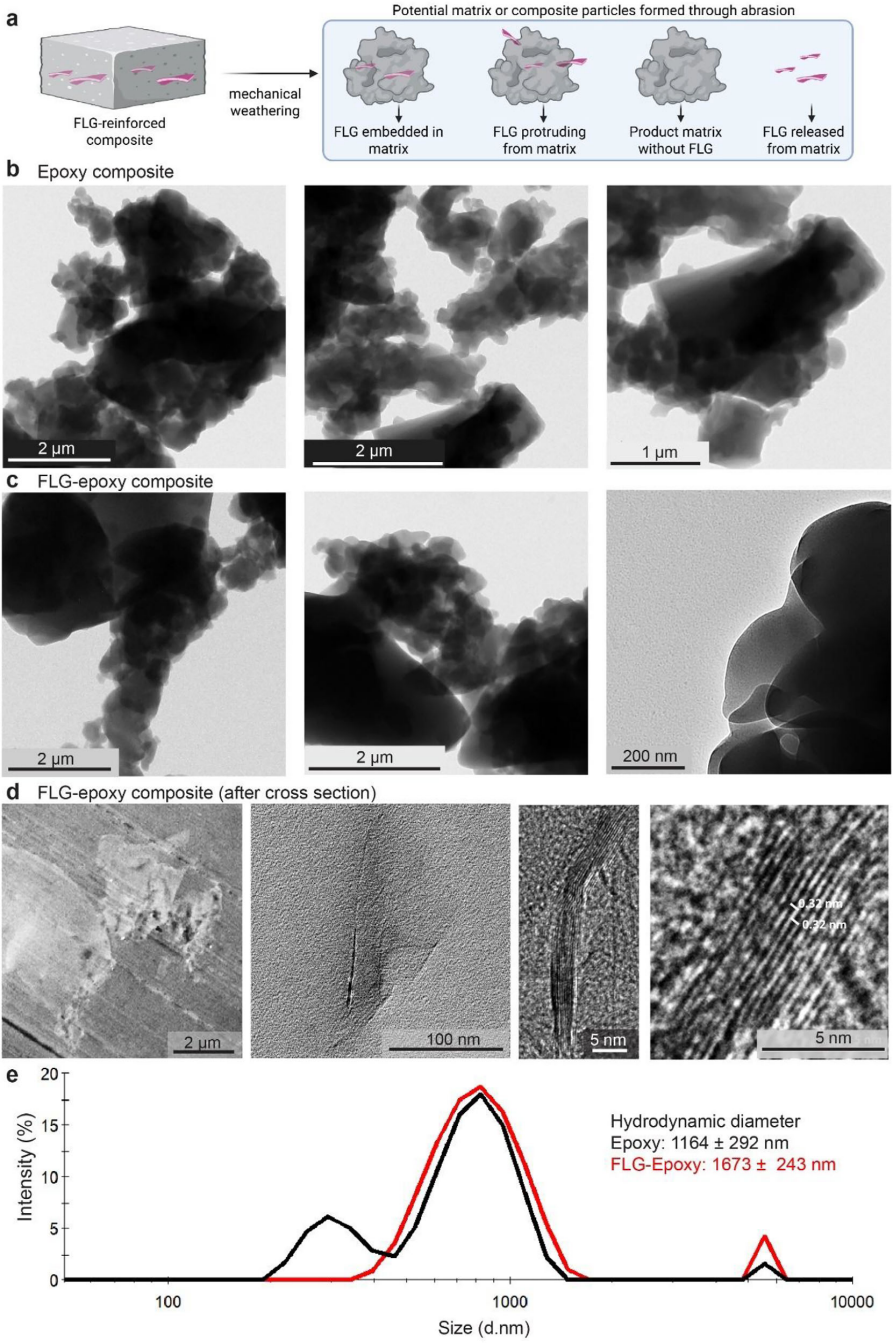
Physical transformation of FLG‐epoxy composite particles produced by mechanical abrasion. (a) Sketch of potential morphologies of physically transformed composite particles. (b) TEM images of epoxy composites after abrasion. (c) TEM images of FLG‐epoxy composites following abrasion. (d) HR‐TEM images of FLG‐epoxy cross‐section, indicating FLG embedded within the epoxy matrix. (e) Hydrodynamic diameter measurements with mean ± SD of abraded epoxy and FLG‐epoxy composites dispersed in 0.1% BSA‐water after 45 min bath sonication.

ToF‐SIMS has previously been applied for the characterization of additives in polymer‐based commercial products [35]. Motivated by these findings, we explored whether ToF‐SIMS could likewise detect few‐layer graphene (FLG) and possible contaminants in epoxy composites. However, whereas SIMS is frequently used for the detection of contamination in polymeric systems [36, 37], the detection of graphene by ToF‐SIMS has turned out to be challenging. While graphene and graphene oxide are increasingly employed as matrices to enhance the detection of other analytes [38, 39], direct detection of graphene itself remains difficult [40].

Analysis of carbon allotropes using conventional ToF‐SIMS, i.e., with bismuth primary ions and a time‐of‐flight detector, typically yields only comparatively small and unspecific carbon clusters [41]. We have observed that graphene analysis of pure material samples using so‐called Orbi‐SIMS, i.e. argon cluster bombardment in combination with an Orbitrap detector can yield high‐mass graphene fragments (data not shown). Nevertheless, Orbi‐SIMS analysis of unpolymerized epoxy samples, both containing and lacking graphene at the target concentrations, did not reveal signals characteristic of graphene. The same observation was made for polymerized samples before as well as after abrasion. In summary, an unambiguous identification of graphene in the epoxy samples was not successful as its concentration in the probed volume appears to be below the detection limit under the selected conditions.

Given the complexity of the carbon‐rich epoxy matrix, we searched for indirect or proxy signatures that could indicate the presence of FLG. Although graphene itself could not be detected unambiguously by ToF‐SIMS, however, the CNO^−^ and Cl^−^ signals exhibited no measurable contribution from the epoxy background (Figure 3) but showed spatial distribution patterns closely matching the FLG features observed in the Raman 3D depth profiles (Figure 2). These observations suggest that while ToF‐SIMS does not directly detect FLG in this system, it can provide useful localization of FLG‐associated regions.

Furthermore, we also examined whether contaminants were introduced at different stages of composite processing, namely in the unpolymerized precursor material, in the fabricated composite plates, and in the particles released by mechanical abrasion. Previous studies have demonstrated that ToF‐SIMS is well‐suited for identifying such impurities in graphene–polymer systems. Brennan et al. (2017) [42], for example, detected a range of inorganic contaminants (Na, Li, Mg, K, Fe, F, I, Br, Ca, S, and Cl) in graphene–poly(acrylic) nanocomposites, largely originating from the surfactant used during fabrication. Likewise, Michałowski et al. (2022) [43] reported the presence of sodium and sulfur on the surface of commercial graphene materials using ToF‐SIMS.

Consistent with these observations, the Orbi‐SIMS spectra of our epoxy and FLG–epoxy plates revealed several potential impurities, including alkylbenzene sulfonate (C_18_H_29_SO_3_^−^) and palmitate (C_16_H_31_O_2_^−^) (Figure S1). Notably, lauryl sulfate (C_12_H_25_SO_4_^−^), a common surfactant molecule, was detected exclusively in the FLG‐epoxy plates (Figure S1b). Depth profiling further showed that these impurities were concentrated on the outermost surface layers of the composite plates (Figure S2), suggesting that at least part of the contamination may have been introduced during handling after fabrication. Such impurities were also detectable, albeit at very low intensities, in the abraded particles, which may be relevant for toxicological considerations when assessing the release and exposure potential of FLG‐epoxy composites.

Mechanical abrasion of solid FLG‐epoxy polymer composites at the end‐of‐life could release physically transformed particles in the micro and/or nano size range, with FLG either fully covered by the polymer matrix or protruding from the surface as illustrated in Figure 4a. It is also possible that some fragmented particles may not contain embedded FLG, and in rare cases FLG might be released from the matrix as shown and discussed elsewhere for other carbon materials (e.g., carbon nanotubes) [23, 24, 44]. To investigate potential physical transformations, HR‐TEM imaging of abraded epoxy and FLG‐epoxy composites was performed. As shown in Figure 4b–d, the most prominent abraded composite particles contain FLG enclosed within the epoxy matrix. To confirm the matrix enclosure of FLG, we inspected cross‐sections of composite particles (Figure 4d). Free FLG flakes or FLG flakes protruding from the matrix were not observed. We further showed that the detected FLG in the cross‐sections consisted of 10–12 layers, with a step height between the layers being approximately 0.32 nm (Figure 4d), confirming the typical signature of the reference FLG material (Figure 1b). It is important to note that we did not observe the release of free FLG from the epoxy composites. However, this may be due to the detection limits of the instruments used, as the quantity of free FLG would likely be extremely low and difficult to locate within such a complex matrix.

Nevertheless, safety concerns of physically transformed composites produced by mechanical abrasion still need to be addressed carefully, since these fragmented composites are transformed materials that could behave differently in biological environments compared to their corresponding pristine counterparts [45]. Moreover, in some cases, structural transformations of the composites released after mechanical breakdown or combustion have been shown to pose safety concerns. For example, inhalation of composites or particles with sharp protrusions could cause mechanical damage in cells once the composites enter the respiratory tract, as has been evidenced for fibers and graphene microsheets [46, 47]. On the other hand, the transformation of materials in the product matrix could also modulate their toxicity as compared to their pristine counterparts. Previous studies have demonstrated that inhalation exposure to nanocomposites reinforced with graphene or other 2D materials did not trigger acute effects in lung, immune and skin cells [12, 22, 48, 49, 50]. Considering the limited inhalation exposure studies on advanced materials under simulated end‐of‐life conditions, we next examined whether epoxy and FLG‐epoxy particles released from mechanical abrasion pose any hazard to lung cells.

For experiments with lung cell cultures, the size of the abraded particles in suspension was characterized by DLS. The average hydrodynamic size of abraded epoxy and FLG‐epoxy particles in water was determined to be 1164 ± 292 nm and 1673 ± 243 nm (± SD), respectively (Figure 4e). However, DLS has important limitations for the characterization of irregular, high‐aspect‐ratio, and highly polydisperse abraded fragments because the technique derives a hydrodynamic diameter based on Brownian motion and an assumed spherical particle geometry. The DLS‐derived sizes should therefore be interpreted as complementary information on particle behavior in suspension rather than as a representation of the aerodynamic particle‐size distribution. Additional real‐time measurements during abrasion detected an airborne submicrometer particle population with electrical mobility diameters predominantly between approximately 40–400 nm (Figure S3). Because particles above approximately 1 µm were removed upstream of the instrument, these measurements demonstrate the presence of a submicrometer airborne fraction but do not represent the complete abrasion‐particle size distribution or allow quantification of the submicrometer fraction relative to larger particles. Consistent with this limitation, analysis of the filter‐collected material revealed a small number of larger particles, which were removed for selected experiments with macrophage cultures (see below). The endotoxin levels in abraded epoxy and FLG‐epoxy composites suspension (100 µg/mL) were 0.002 ± 0.001 EU mL^−1^, and 0.013 ± 0.008 EU mL^−1^, respectively, which are well below the United States Food and Drug Administration threshold limit (<0.5 EU mL^−1^), suggesting that both particles were endotoxin‐free.

### 2.3 Cytotoxicity of flg and Abraded FLG‐Epoxy Composites and Their Effects on Mucociliary Clearance Function in Primary Bronchial Epithelial Cell Cultures

The bronchial epithelium is the first contact point after inhalation of pollutants, including abraded composite particles. Physiologically, the bronchial epithelium is covered by beating cilia (at the surface of ciliated cells) and a layer of respiratory mucus (secreted by goblet cells) (Figure 5a). The mucus is a gel‐like structure composed mainly of glycoproteins (mucins) that can trap most incoming pollutant particles, which then get cleared by conducting cilia (defined as mucociliary clearance). However, particles that can escape mucociliary defenses can reach the epithelium, potentially affect the epithelial barrier and trigger adverse effects. Certain nanomaterials have been shown to impair mucociliary defenses and cause damage to bronchial epithelial cells [51] and are even considered a leading cause of lung fibrosis. Therefore, we investigated whether exposure to FLG and abraded epoxy composites affects the bronchial epithelium and its mucociliary clearance functions. To this end, we used reconstituted primary human bronchial epithelial (pHBE) cell cultures that develop moving cilia, secrete mucus and form a tight epithelial barrier as illustrated in Figure 5a and characterized them by immunofluorescence staining (Figure 5b). The confocal images showed intact tight junctions (indicated by ZO‐1 staining) and the presence of cilia (indicated by anti‐tubulin staining) on the surface of ciliated cells (Figure 5b and Figure S4). In addition, repeated exposure (once a day for four consecutive days) to abraded epoxy and FLG‐epoxy composites or as‐produced FLG had no measurable impact on the tight junctions, suggesting an intact epithelial barrier (Figure 5b and Figure S4). However, we observed a slight but significant (*p*<0.05) decrease in the transepithelial electrical resistance (TEER) after 2 days of exposure to FLG at 10 and 30 µg cm^2^ with respect to the vehicle control (0.1% BSA), which normalized after 4 days (Figure 5c). Similarly, abraded epoxy composites induced a significant decrease in TEER value after 2 days of exposure at 50 and 100 µg cm^2^, which normalized at day 4 (Figure 5d). In contrast, abraded FLG‐epoxy composites induced an increase in TEER after exposure to 10 and 50 µg cm^2^ at days 1 to 4, whereas 100 µg cm^2^ triggered a significant decrease by day 4 (Figure 5e). It is important to note that TEER values in all exposure conditions were still above the threshold of 100 Ω × cm^2^, which indicates no disruption of the epithelial barrier [51]. Collectively, these results suggest that the transient changes in TEER might reflect a treatment‐related deregulation in membrane ion channels rather than a disruption of barrier integrity.

**FIGURE 5:**
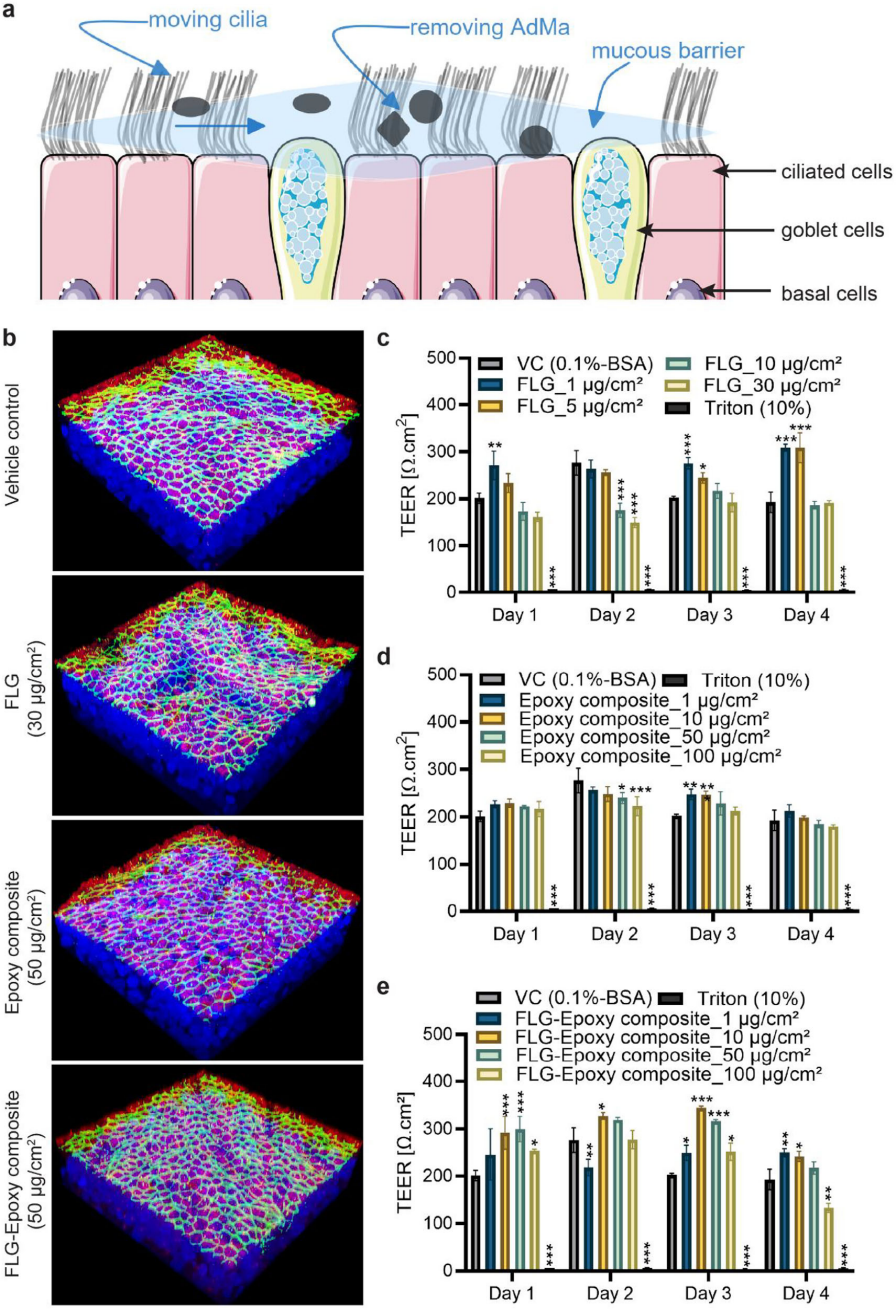
Epithelial barrier integrity of primary bronchial epithelial cell cultures (MucilAir^TM^) after repeated exposure to FLG or abraded FLG and epoxy composites. (a) Schematics showing the mucociliary clearance (MCC) function (e.g., removing potentially inhaled advanced materials, AdMa) of bronchial epithelial cells in the respiratory airway. (b) Confocal images showing no effect on the distribution of tight junction protein ZO‐1 staining (green) after 4 days of repetitive exposure to the indicated particles. Cell nuclei and respiratory cilia are stained in blue (DAPI) and red (tubulin), respectively. (c–e) Time‐dependent effect on TEER in pHBE cell cultures after exposure to (b) FLG, (c) abraded epoxy composite and (d) abraded FLG‐epoxy composite. Data are presented as mean ± SD (*n* = 3). Each cell culture was reconstituted from primary cells pooled from five different donors. Statistical significance was calculated between vehicle control (VC) and individual treatments by applying two‐way ANOVA with Dunnett's multiple comparisons test. *p<0.05; **p<0.01; ***p<0.001.

We next investigated whether exposure to FLG or abraded epoxy and FLG‐epoxy affects ciliary functions or induces changes in mucociliary clearance (Figure 6). As shown in Figure 6a–c, no significant effects were observed in cilia beating frequency (CBF) after 4 days of exposure to abraded epoxy and FLG composites or FLG alone. The mucociliary clearance (MCC; indicating clearance of polystyrene beads by conducting cilia after 4 days of exposure) was slightly decreased in a dose‐dependent manner after exposure to abraded epoxy composites with respect to the vehicle control (0.9% NaCl) (Figure 6e). However, FLG or abraded FLG‐epoxy composites had no significant effects on the MCC (Figure 6d–f), suggesting epoxy matrix rather than FLG is the main driver. Further, SEM analysis revealed an accumulation of particle‐secreted mucus complexes on the surface of pHBE cell cultures (in the ciliary region) compared to untreated control cells, further confirming a decreased MCC function of the cell cultures after 4 days of exposure (50 µg cm^2^) to abraded epoxy composites (Figure 6g and Figure S5). However, ciliary morphology was not disturbed as visible in SEM images after exposure to either abraded epoxy and FLG composites or FLG alone. Chortarea et al. [52] previously demonstrated a reduced MCC function of asthmatic pHBE cell cultures following repetitive exposure (5 days per week) to MWCNTs (10 µg cm^2^) over 5 weeks. Exposure to silicon carbide nanowires has also been reported to alter ciliary morphology and impair MCC function, whereas no effects were observed for graphene nanosheets, MWCNTs, silicon dioxide nanoparticles, or silica quartz [51]. Similarly, Beyeler et al. found no impairment of ciliary function in pHBE cultures derived from healthy donors or COPD patients following short‐term (24 h) exposure to MWCNTs [53]. *In vivo* studies further demonstrated that inhalation of certain nanomaterials (primarily copper oxide nanoparticles, carbon nanotubes, and boron nitride nanotubes) can promote hyper‐mucus secretion in mice, resulting in bronchial wall thickening [54, 55, 56].

**FIGURE 6:**
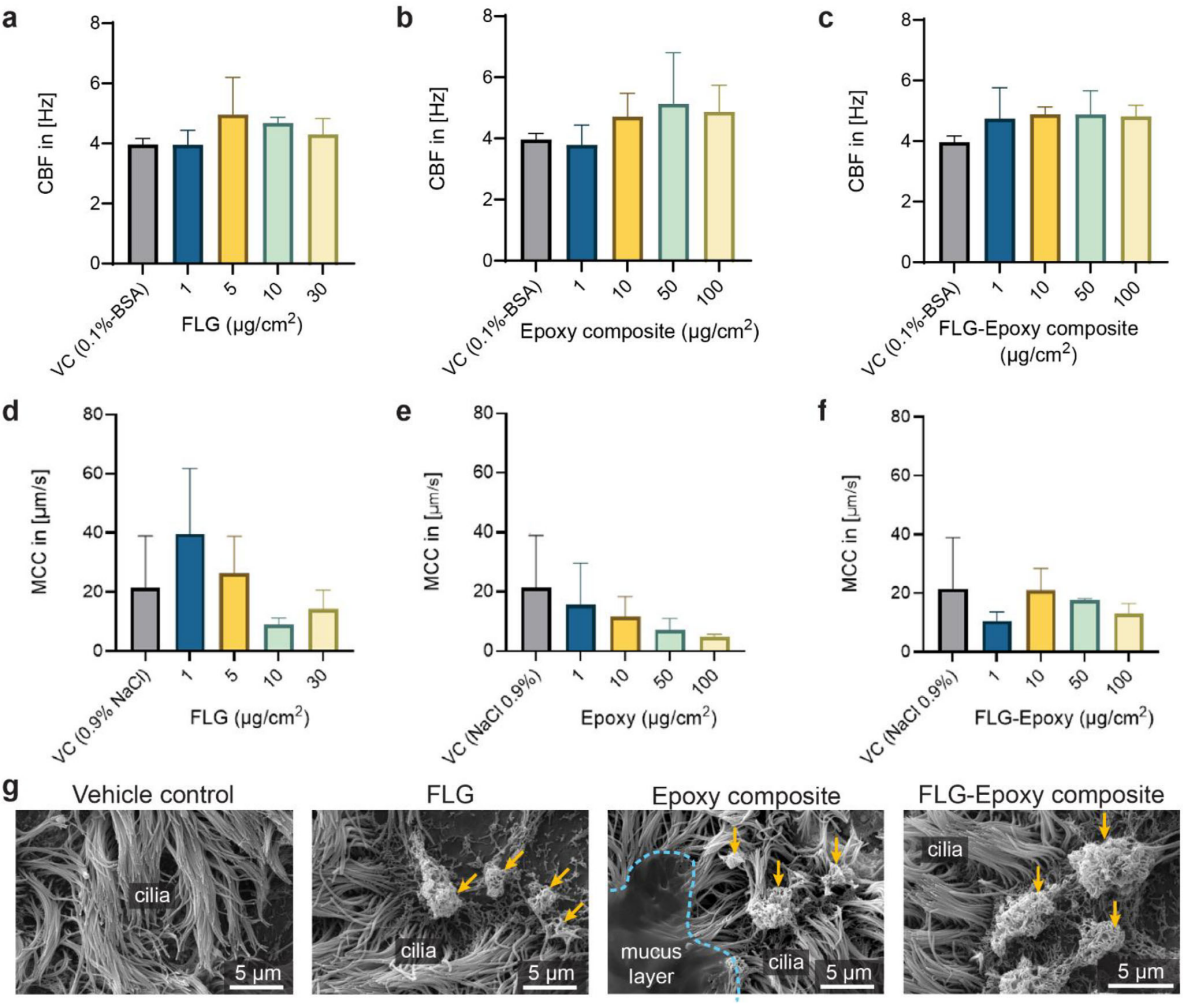
Functional effects on MCC after exposure to abraded epoxy and FLG‐epoxy composites following 4 days of repetitive exposures at indicated doses. (a–c) No significant effects were recorded in the cilia beating frequency after 4 days of exposure to FLG (a), abraded epoxy composite (b), and (c) abraded FLG‐epoxy composite. (d–f) MCC function of pHBE cell cultures after exposure to FLG (d), abraded epoxy composite (e), and abraded FLG‐epoxy composite (f). Data presented in (a‐f) are mean + SD (*n* = 3). (g) SEM images of pHBE cell cultures (see also Figure S4) further confirmed a reduced clearance of mucus and trapped materials compared to the untreated control after repeated exposure to indicated materials at 30 µg/cm^2^ for 4 days, but no significant changes to ciliary morphology. Yellow Arrows: mucus‐particle aggregates; dotted cyan lines: uncleared mucus.

We next asked whether the accumulation of large mucus‐particle complexes on the surface of pHBE cell cultures (Figure 6g) could induce cytotoxicity or inflammatory responses in the epithelium. As shown in Figure S6a–c, no significant (*p*>0.05) increase in LDH release (representing cell membrane rupture) was observed after day 2 and day 4 of exposure to abraded epoxy and FLG‐epoxy composites or FLG alone at the tested dose range. Similarly, the secretion of the inflammatory cytokine interleukin (IL)‐8 remained unaffected across all conditions and time points. To further assess immune responses, we screened the cell culture supernatant using a 48‐plex cytokine‐chemokine array after 4 days of repetitive exposure to FLG or abraded epoxy and FLG‐epoxy composites (10 µg cm^2^). FLG significantly (*p*<0.05) induced the release of inflammatory cytokines IL‐1RA, IL‐1α, sCD40L, IL‐6 and fibroblast growth factor (FGF)‐2 with respect to vehicle control (0.1% BSA‐water; Figure S7). Abraded epoxy composites induced release of inflammatory cytokines interferon‐gamma‐inducible protein (IP)‐10 (a/k CXCL10), macrophage colony‐stimulating factor (M‐CSF), RANTES, granulocyte colony‐stimulating factor (G‐CSF), monocyte chemotactic protein (MCP)‐1 and macrophage‐derived chemokine (MDC). However, no significant (*p*>0.05) effects in cytokine‐chemokine secretion were evident after exposure to abraded FLG‐epoxy composites when compared to vehicle control.

Overall, these results suggest that abraded FLG‐epoxy composites do not trigger acute cytotoxic effects at the indicated dose range in pHBE cell cultures. However, abraded epoxy composites may reduce the MCC function but reinforcing them with FLG does not further exacerbate this effect.

### 2.4 Cytotoxicity and Immunomodulatory Response of Alveolar Lung Cell Cultures to Abraded FLG and Epoxy Composites

It has recently been confirmed that alveolar macrophages play a key role in initiating (nano)particle‐induced pulmonary toxicity and immunomodulatory effects (e.g., neutrophil recruitment, cytokine‐chemokine responses) in vivo [57]. Therefore, we asked whether exposure to FLG, or abraded epoxy and FLG‐epoxy composites induces cytotoxic and immunomodulatory effects in alveolar macrophages. To this end, the alveolar macrophage assay based on NR8383 cells was employed using the standard dose range of 22.5, 45, 90, and 180 µg mL^−1^ (corresponding to 14, 28, 56, and 112 µg cm^2^) [58].

To verify cellular uptake of FLG, abraded epoxy and abraded FLG‐epoxy in the cells, CRM analysis was performed, and 3D reconstructions were generated using particle‐specific FLG or epoxy Raman signatures within the mapped regions (Figure 7a,b and Videos S2–S4). The results demonstrated the presence of FLG within alveolar macrophages exposed to either FLG or abraded FLG‐epoxy composite. In contrast, only epoxy was detected inside cells exposed to abraded epoxy composites. Furthermore, correlative imaging of the same cells by SEM following CRM analysis revealed not clearly distinguishable FLG, epoxy, or FLG–epoxy particles on the cell surface, suggesting that the FLG or epoxy signals observed in CRM images originated from intracellular material (Figure 7c).

**FIGURE 7:**
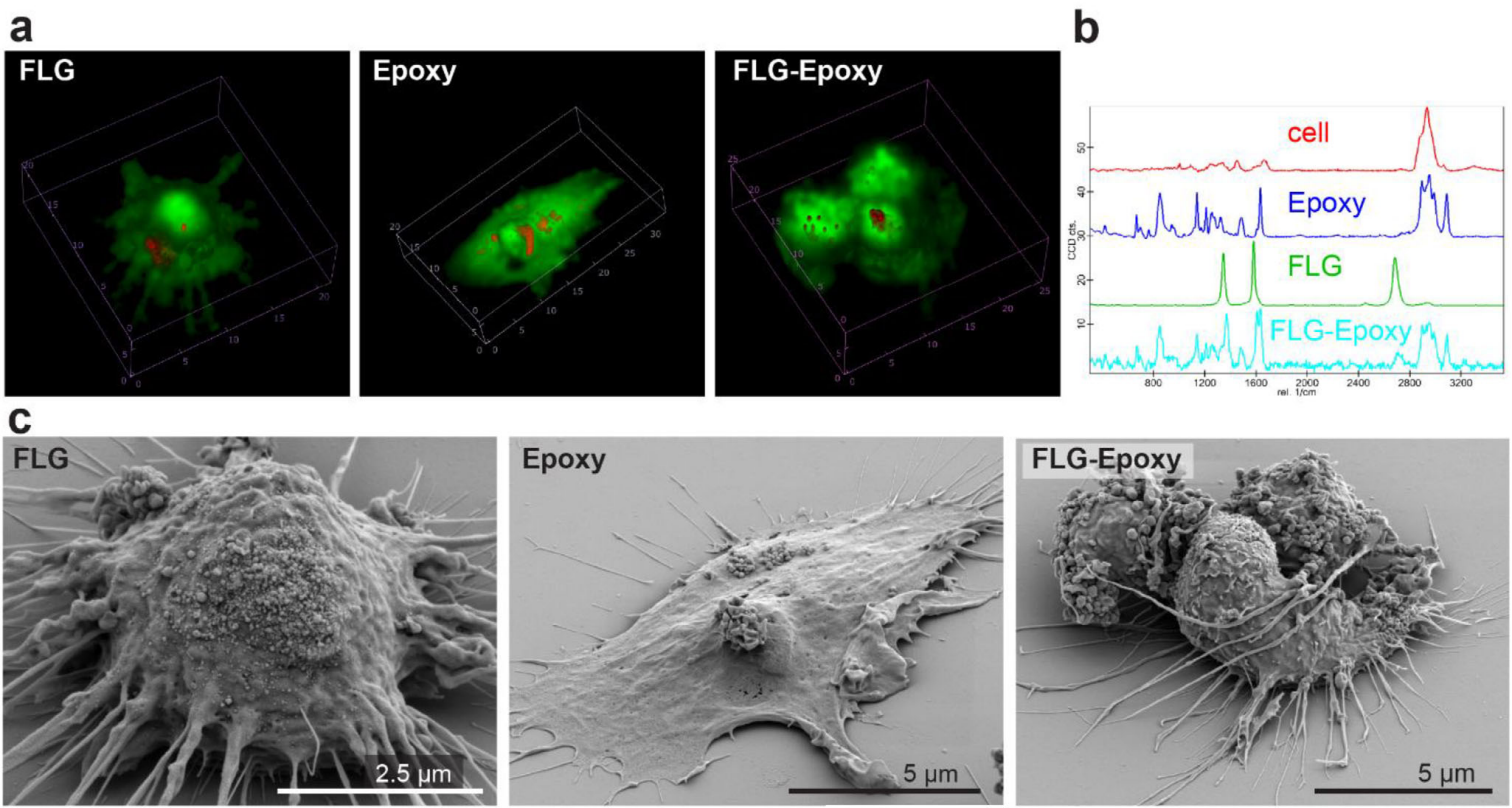
Correlative microscopy images of particle‐loaded NR8383 alveolar macrophages viewed with Confocal Raman Microscopy (CRM) followed by Scanning Electron Microscopy (SEM). Particles outside cells were removed by extensive washing. Scale bars: 20 µm. (a) CRM 3D image stacks loaded with 11.25 µg mL^−1^ of either FLG, abraded epoxy or abraded FLG‐epoxy material as indicated. Particles (red) are located inside the cells (green). (b) Reference Raman spectra of alveolar macrophage cells (red), FLG (green) and abraded epoxy (blue) or FLG‐epoxy (cyan). (c) Corresponding SEM micrographs of cells shown in (a).

Microscopic inspection of the particle‐treated cells during exposure experiments showed that some particles of both abraded materials were large (> 10 µm) and not engulfed by the macrophages. To remove these particles, the aqueous particle suspensions were passed through a 5 µm gauze such that as‐is and filtrated particles could be tested side by side and compared with FLG. In FLG‐treated cells, dark intracellular inclusions were observed within the cell boundaries (Figure 8a). In contrast, alveolar macrophages exposed to the original (non‐fractionated) epoxy or FLG–epoxy particles exhibited heterogeneous and incomplete loading: some cells contained or were associated with large particulates, while others showed no evident particle uptake. By comparison, the size‐fractionated epoxy (≤ 5 µm) and FLG–epoxy (≤ 5 µm) particles were internalized by the majority of cells, resulting in a more uniform and homogeneous particle distribution across the cell population.

**FIGURE 8:**
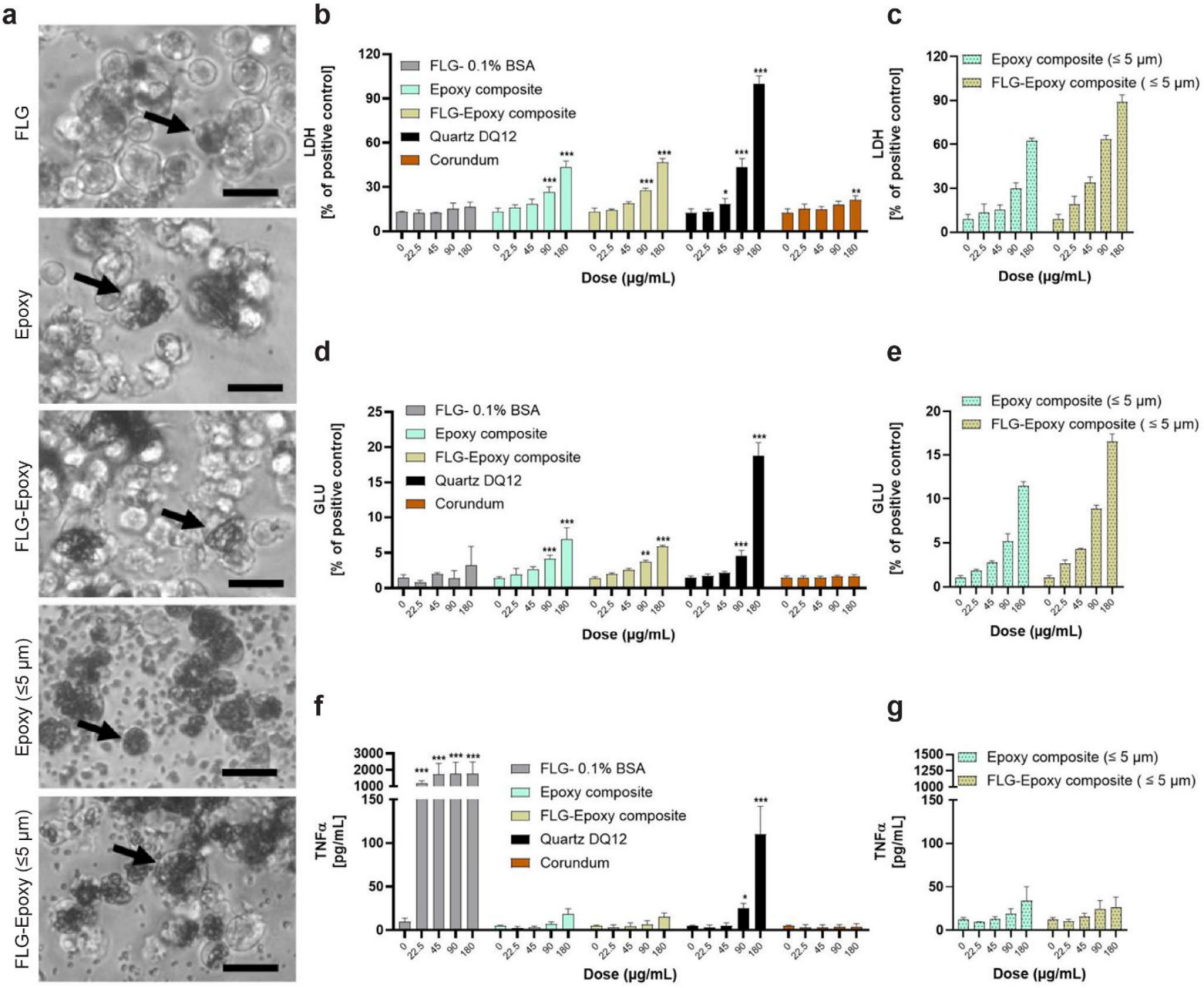
Cytotoxicity and inflammatory responses induced by FLG, abraded epoxy and abraded FLG‐epoxy composites in NR8383 alveolar macrophages after 16 h of exposure in serum‐free cell culture medium. Size‐fractionated abraded materials lacking large particles (epoxy ≤ 5 µm; FLG–epoxy ≤ 5 µm) were tested in parallel. Corundum and quartz DQ12 served as negative and positive controls, respectively. (a) Phase contrast images of NR8383 cells exposed to 180 µg mL^−1^ of each particle type. FLG‐treated cells exhibit dark intracellular inclusions (arrow). Cells exposed to abraded epoxy or FLG–epoxy are associated with larger particles (arrows), whereas cells treated with epoxy ≤ 5 µm or FLG–epoxy ≤ 5 µm show a homogeneous distribution of small particles. Scale bars: 20 µm. (b–g) The following enzymes and mediators were measured in the cell culture supernatant after 16 h of treatment: (b,c) lactate dehydrogenase (LDH) to indicate membrane damage, (d,e) glucuronidase (GLU) to indicate lysosomal damage and (f,g) TNF‐α to indicate inflammatory responses. Epoxy and FLG‐Epoxy composites triggered a limited TNF‐α production at the highest indicated dose. Data presented in (b,d,f) are mean ± SD (*n* = 3). Statistical significance was obtained by applying Two‐way ANOVA and Dunnett's multiple comparison test at *post‐hoc*. The *p*‐value < 0.05 was considered statistically significant. **p < 0.01, ****p < 0.0001 indicates statistically significant with respect to the vehicle control. Data presented in (c,e,g) are mean ± SD of two experiments (due to a limited availability of filtered composite particles) (*n* = 2).

FLG did not induce cytotoxicity, with lactate dehydrogenase (LDH) and glucuronidase (GLU) release comparable to the corundum (negative control). However, the release of LDH and GLU was identical for both types of abraded epoxy and FLG‐epoxy composite particles, indicating a similar dose‐dependent cytotoxicity (Figure 8b,d). As expected, the size‐limited fractions of both types of particles elicited an augmented release of LDH and GLU, which was, however, slightly increased for the abraded FLG‐epoxy composite particles (Figure 8c,e). Since FLG alone had no cytotoxic effect, we suggest that the observed effects of abraded epoxy and FLG‐epoxy composite particles were mainly driven by the epoxy matrix, with no direct contribution from FLG present in the epoxy matrix. The slight differences between the response to abraded epoxy and FLG‐epoxy composite particles may, therefore, be attributable to the shape and/or size of the abraded particles, as already measured by DLS (see above). This difference may be interpreted as an indirect effect of FLG influencing the generation of particles during Taber abrasion, e.g. via altered strength or ductility of the composite material.

Next, we investigated the release of TNF‐α from cells to understand the inflammatory response after exposure to abraded FLG and composites. As shown in Figure 8f, the exposure to FLG alone elicited a very high TNF‐α production at all the indicated doses, which exceeded the LPS response. In contrast, abraded epoxy and FLG‐epoxy composites triggered a moderate dose‐dependent TNF‐α response even at the highest exposure dose (180 µg mL^−1^). As outlined above, we also showed that the FLG nanosheets were endotoxin‐free. Furthermore, the FLG‐induced TNF‐α expression was not inhibited by polymyxin B (Figure S8). Therefore, the TNF‐α production upon FLG exposure was regarded as a particle effect rather than an endotoxin contamination. Interestingly, the abraded FLG‐epoxy composites did not increase the TNF‐α production in an over‐proportional manner for both non‐filtered and filtered particles (Figure 8f,g), suggesting that the vast majority of the FLG nanosheets were fully embedded and covered by an epoxy matrix, as found by HR‐TEM images (Figure 4). The pronounced pro‐inflammatory response observed following exposure to FLG may be influenced by changes in surface chemistry occurring during material processing and dispersion. In particular, partial oxidation of FLG during handling, air exposure, or suspension preparation could affect its interaction with cells. However, the oxidation state of FLG was not characterized in the present study and therefore its contribution to the observed biological responses cannot be determined. Future studies combining detailed surface chemistry characterization with biological testing are needed to establish potential relationships between FLG oxidation state and biological activity.

In line with our findings in rat alveolar macrophages, Chortarea et al. (2022) [12] also showed that reduced graphene oxide (GO) exposure to RAW 264.7 macrophages at 40 µg mL^−1^ for 24 h slightly increased TNF‐α secretion; however, no or limited effects were observed when exposed to abraded PA6 and PA6‐rGO composites. Lin et al. (2020) [59] demonstrated that FLG exposure to M1 and M2 macrophages at 25 and 50 µg mL^−1^ induced TNF‐α production. Cebadero‐Dominguez et al. (2023) [60] showed that rGO exposure in THP‐1 (EC20 = 21.64 ± 0.4 µg mL^−1^) and Jurkat cells (EC20 = 90.83 ± 0.73 µg mL^−1^) at EC20 dose induced expression of inflammatory cytokines, including TNF‐α, after 4 h and 24 h. Song et al. (2025) [61] demonstrated an increase in IL‐1β secretion after exposure to FLG in undifferentiated THP‐1 or THP‐1‐derived macrophages, while no effects were observed in TNF‐α secretion. In contrast, Mukherjee et al. (2018) [62] reported that exposure to GO with small or large lateral dimensions did not trigger TNF‐α or other cytokine‐chemokine secretion in primary human monocyte‐derived macrophages. However, GO exposure to LPS‐primed macrophages increased inflammasome‐dependent IL‐β secretion.

Next, we asked whether exposure to FLG and abraded epoxy or FLG‐epoxy composites can sensitize the alveolar lung cells. To this end, we co‐cultured the alveolar epithelial type II (AT‐II) cells (A549 cell line) and endothelial cells (EA.hy926 cell line) with human macrophages and dendritic‐like immune cell lines (ALIsens model), as illustrated in Figure 9a. The results presented in Figure 9b–d show no cytotoxic effects of FLG or abraded epoxy and FLG‐epoxy composites at the indicated dose range following 24 h of exposure. Next, THP‐1 cells were retrieved from the basolateral compartment of the model after 24 h of exposure and stained against thymic stromal lymphopoietin receptor (TSLPr, potential marker of respiratory sensitization), cluster of differentiation (CD)‐54 (a/k intercellular Adhesion Molecule 1 (ICAM‐1)) and CD‐86 (marker of antigen‐presenting cells). The flow cytometry analysis of these cells showed a significant increase in the expression of TSLPr specifically in the cell cultures exposed to abraded epoxy composites in a dose‐dependent manner (Figure 9f), suggesting potential respiratory sensitization [63]. However, no change in TSLPr expression was observed after exposure to either FLG alone (Figure 9e) or abraded FLG‐epoxy composites (Figure 9g). The lack of TSLPr induction by abraded FLG‐epoxy composites may be explained by the capacity of graphene to adsorb small organic molecules via van der Waals and π–π stacking interactions. Amine‐based hardeners, which can act as sensitizers in their unreacted state [64, 65], have been shown to adsorb onto graphene surfaces [66], suggesting that residual sensitizing species may become sequestered onto FLG within the composite, reducing their bioavailability upon abrasion. While plausible, this remains to be experimentally confirmed.

**FIGURE 9:**
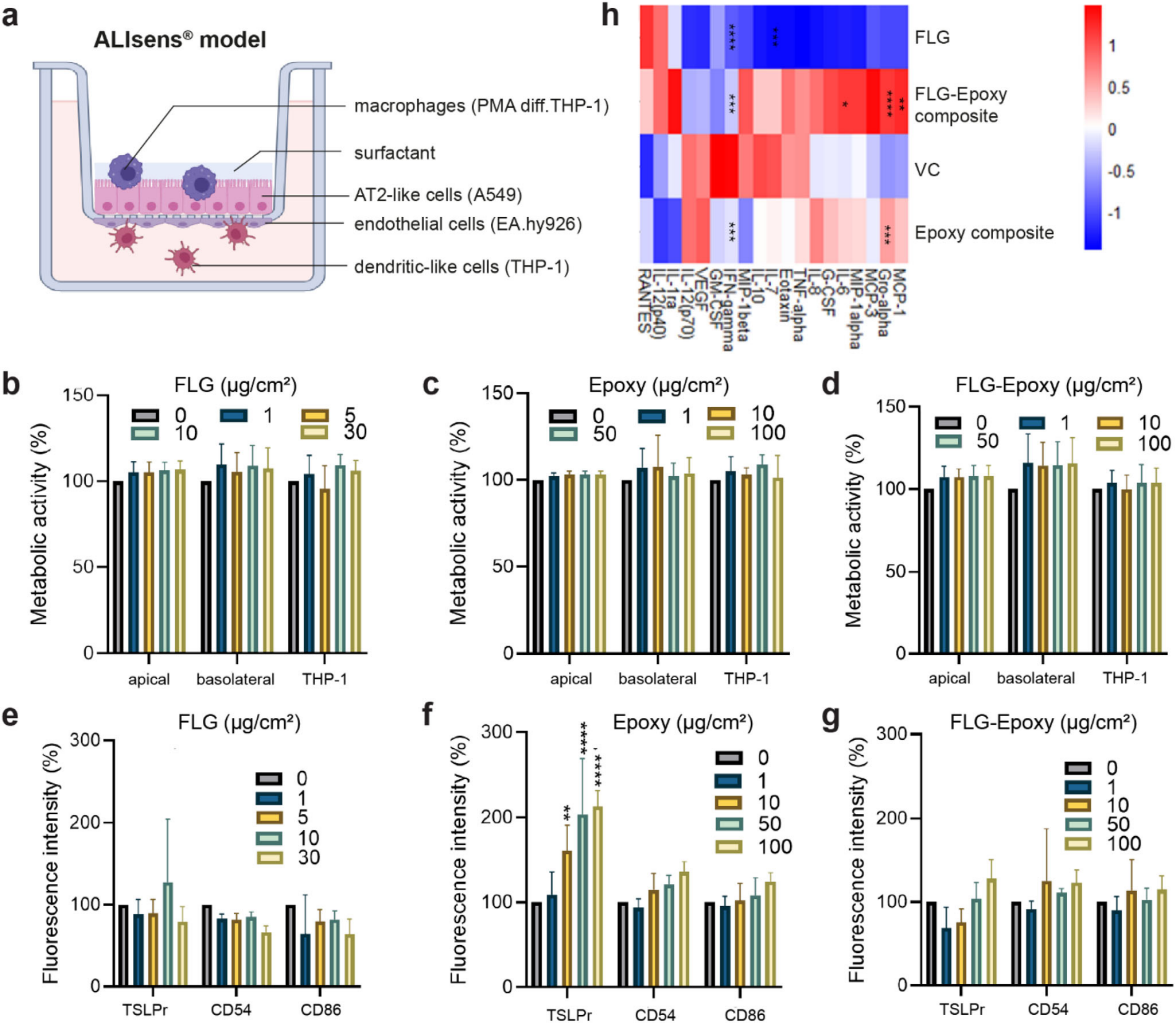
Viability and respiratory sensitization of the ALIsens cell culture model (a) after 24 h of exposure to FLG or abraded epoxy and FLG‐epoxy composites. (b‐d) Metabolic activity (indicating cellular viability) of cells from the different compartments of the model (apical side, basolateral side, and THP‐1 cells) after exposure to (b) FLG, (c) abraded epoxy composites, and (d) abraded FLG‐epoxy composites at the indicated concentrations. (e‐g) Expression of TSLPr, CD54, and CD86 markers on the surface of THP‐1 cells cultured in the ALIsens model after exposure to (e) FLG, (f) abraded epoxy composites and (g) abraded FLG‐epoxy composites at the indicated concentrations. (h) Basolateral release of cytokines and chemokines in the ALIsens model after exposure to FLG alone, abraded epoxy or FLG‐epoxy composites at 10 µg cm^2^. Data presented in the heat map were clustered using mean values (*n* = 4) of detected cytokines and chemokines. Data presented in (b–g) are mean ± SD (*n* = 4). Statistical significance was obtained by applying One‐way ANOVA and Dunnett's multiple comparison test at *post‐hoc*. The *p*‐value < 0.05 was considered statistically significant. **p < 0.01, ****p < 0.0001 indicates statistically significant with respect to the vehicle control (VC; denoted as 0). Data presented in the heat map were clustered using mean values (*n* = 4) of detected cytokines and chemokines. Statistical significance in (h, *n* = 4) was obtained by applying 2‐way ANOVA and Dunnett's multiple comparison test at *post‐hoc*. The *p*‐value < 0.05 was considered statistically significant. *p < 0.05, **p < 0.01, ***p < 0.001, ****p < 0.0001 indicates statistically significant with respect to the vehicle control (denoted as 0).

Furthermore, cytokine‐chemokine release from cell cultures after exposure to FLG, abraded epoxy or FLG‐epoxy composites were investigated in both apical and basolateral compartments; only basolateral results are presented here (Figure 9h). LPS was included as positive control and induced an increase in the analyzed cytokines and chemokines in both compartments, validating the model (data not shown). The FLG‐epoxy exposure induced the release of monocyte chemoattractant protein‐1 (MCP‐1), IL‐6 and growth‐regulated protein (GRO)‐alpha while epoxy induced the release of MCP‐1 and GRO‐alpha. All materials down regulated the interferon‐gamma (IFN‐γ) release. The release of IL‐6, GRO‐alpha and MCP‐1 potentially indicates epoxy and FLG‐epoxy‐induced tissue damage that consequently triggered inflammation and enhanced adhesion of macrophages with lung cells.

Overall, the ALIsens cell culture model offers significant advancements in in vitro screening of respiratory sensitization and immunomodulatory effects of advanced materials composites. However, it is also important to note that the ALIsens model lacks alveolar epithelial type I (AT‐I) cells, which are among the most abundant cell types in the distal lung, covering 95% of the alveolar surface area [67, 68].

We next used the AlveolAir cell cultures reconstituted using primary human epithelial (AT‐I and AT‐II) and endothelial cells from alveolus tissue (Figure 10a). Cellular uptake of epoxy and FLG‐epoxy particles was confirmed by TEM (Figure S9). As shown in Figure 10b–d, repeated exposure of FLG alone and abraded epoxy composites at indicated concentrations for 4 days did not trigger a significant reduction in TEER value with respect to vehicle control, suggesting intact epithelial barrier integrity. However, a significant (*p*< 0.05) reduction in TEER value was recorded in cell culture exposed to abraded FLG‐epoxy composites at day 3 (50 µg cm^2^) and day 4 (50 and 100 µg cm^2^). Again, the reduced TEER values remained above the threshold of 100 Ω×cm^2,^ which is considered indicative of a compromised epithelial barrier, as previously discussed for the pHBE cell culture. Furthermore, no increase in LDH release (reflecting cytotoxicity) was observed after day 2 and day 4 of exposure to either FLG or abraded epoxy and FLG‐epoxy composites at the indicated concentration (Figure S10). IL‐8 release was significantly (*p*<0.05) increased after 4 days of exposure to FLG (5 µg cm^2^) and abraded FLG‐epoxy composites (10 µg cm^2^) compared to vehicle control (0.1% BSA); however, no significant effects were observed after exposure to abraded epoxy composite (Figure 10e–g). Additionally, no significant increase in IL‐8 release was observed after 2 days of exposure.

**FIGURE 10:**
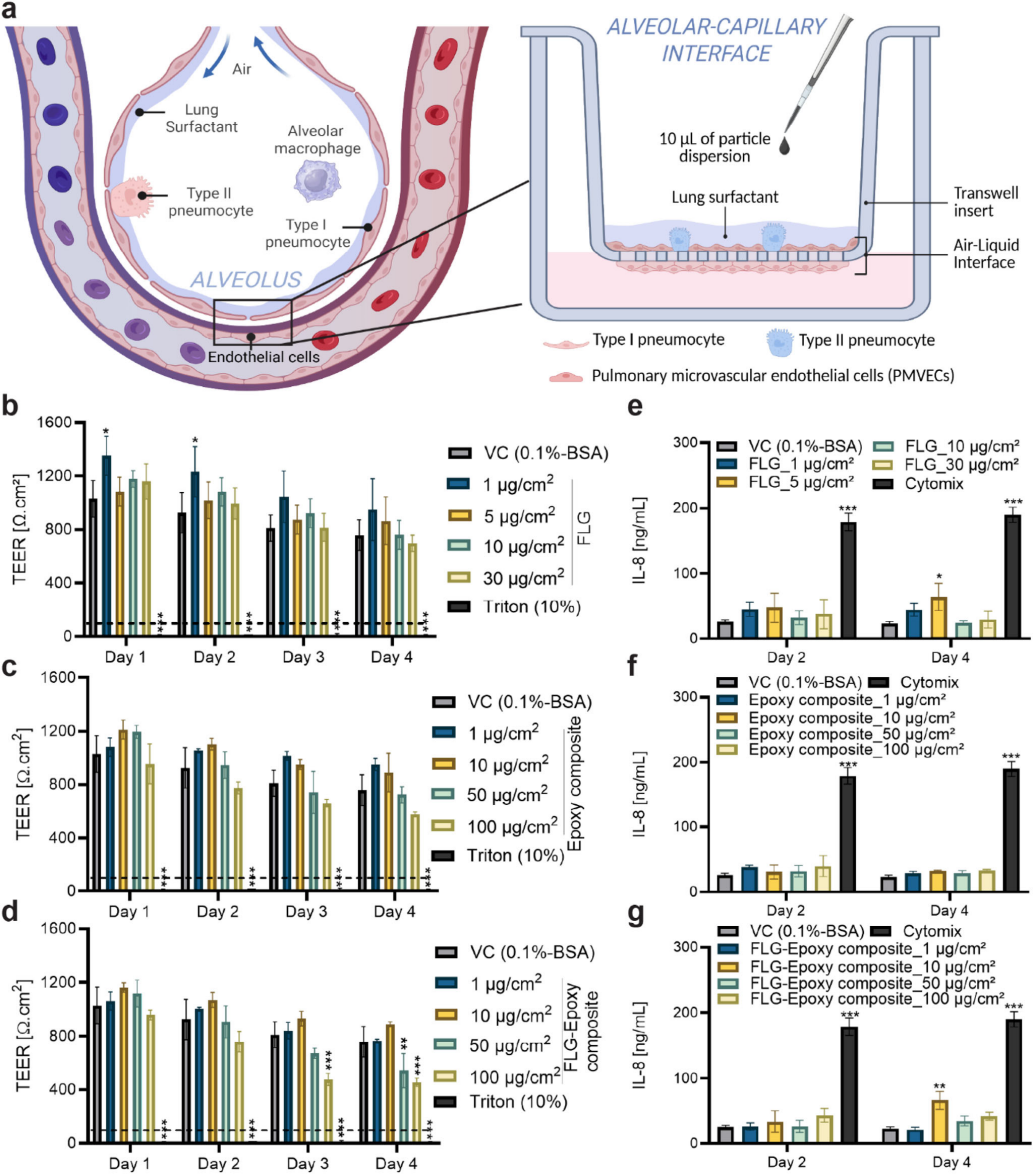
Epithelial barrier integrity and inflammatory response of primary alveolar epithelial cell cultures (AlveolAir^TM^) after repeated exposure to FLG or abraded epoxy and FLG‐epoxy composites for up to 4 days. (a) Illustration showing the AlveolAir^TM^ cell culture model, consisting of type I and type II pneumocytes and endothelial cells, and maintained under ALI conditions. Exposure to FLG nanosheets and abraded composite particles was performed by adding 10 µL of dispersed particles onto the apical surface. The exposure was repeated every 24 h up to 4 days. (b,c) TEER values (indicating transepithelial barrier resistance) of cell culture after exposure to (b) FLG, (c) abraded epoxy composites, and (d) abraded FLG‐epoxy composites at indicated concentrations. LDH release (indicating cytotoxicity response) results are provided in Figure S9. (e–g) IL‐8 release (indicating inflammatory response) from cell cultures after exposure to (e) FLG, (f) abraded epoxy composite and (g) abraded FLG‐epoxy composite at the indicated concentrations and time points. Positive controls: 10% Triton X‐100 or CytoMix. Data are presented as mean ± SD (*n* = 3). Statistical significance was calculated between vehicle control (VC, 0.1% BSA) and individual treatments by applying two‐way ANOVA with Dunnett's multiple comparisons test. The *p*‐value < 0.05 was considered statistically significant. *p < 0.05, **p < 0.01, ***p < 0.001, ****p < 0.0001 indicates statistically significant with respect to the vehicle control (denoted as VC).

The IL‐8 results suggested that FLG or abraded FLG‐epoxy composites may induce a mild inflammatory response in the cells. To further explore immunomodulatory effects of FLG and abraded FLG‐epoxy composites in alveolar cell culture, we performed cytokine‐chemokine screening using a 48‐plex array. As shown in Figure 11, we observed a significant increase in the release of a range of cytokines in positive controls (cells exposed to CytoMix). However, no major changes were detected in the release of cytokines‐chemokines after exposure to FLG or abraded epoxy and FLG‐epoxy composites. In fact, the release of some inflammatory cytokines (PGDF‐AA, FGF‐2, IFN‐a, TGF‐a, IL‐6 and IL‐1β) was significantly decreased with respect to the vehicle control (0.1% BSA) after exposure to FLG‐epoxy composites. We observed a slight increase in the release of IL‐3, G‐CSF, IL9, IL7. GRO‐alpha, MCP3 and Eotaxin after exposure to FLG, but results were not statistically significant due to high inter‐assay variability as plotted in Figure S11. Taken together, the IL‐8 (Figure 10g) and multiplex array data (Figure 11 and Figure S11) suggest that exposure to abraded FLG‐epoxy composites may induce a limited inflammatory response following short‐term repeated exposure (4 days) at a relatively high dose (10 µg cm^2^). The increase in release of IL‐8 and GRO‐alpha was also observed in ALIsens cell culture model as discussed above (Figure 9h), suggesting potential reproducibility of results across different models. These effects warrant attention and highlight the need for further investigations into potential long‐term chronic effects at occupationally relevant exposure levels.

**FIGURE 11:**
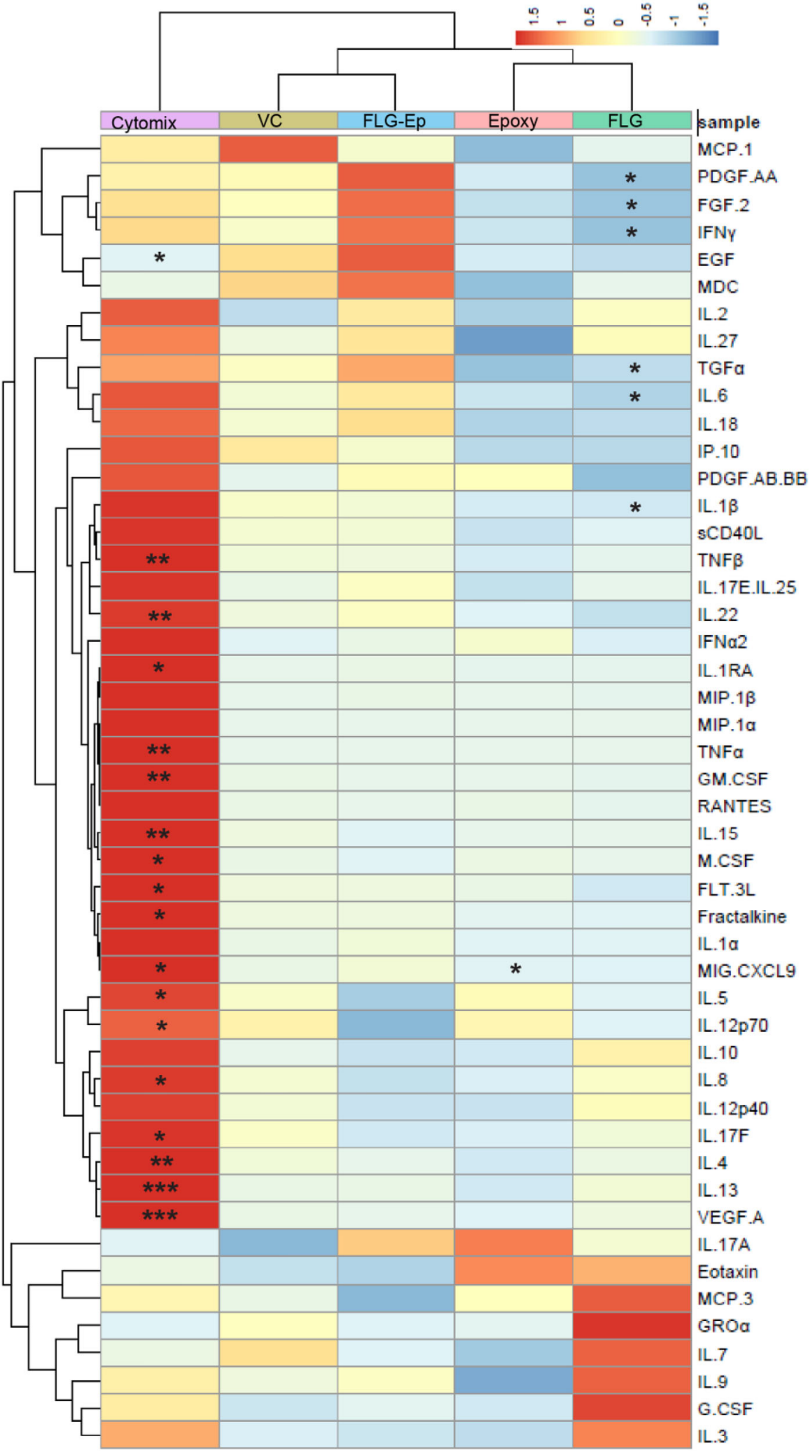
Cytokine‐chemokine responses of primary alveolar cell cultures after 4 days of repetitive exposure to FLG alone or abraded epoxy and FLG‐epoxy composites at 10 µg cm^2^. Data presented in the heat map were clustered using mean values (*n* = 3) of detected cytokines and chemokines. Statistical significance was calculated between individual treatments *versus* vehicle control by applying a T‐test. A *p*‐value < 0.05 was considered statistically significant. *p < 0.05, **p < 0.01, ***p < 0.001, indicates statistically significant with respect to the vehicle control (denoted as VC).

In previous studies, Cristo et al. 2020 [69] demonstrated that repeated exposure to aerosolized graphene oxide for four weeks (maximum delivered dose 20 µg cm^2^) in 3D human bronchial tissue enhanced TNF‐α and IL‐1β production. In contrast, Drasler et al. (2018) [70] have shown that a single exposure to graphene oxide and graphene nanoplatelets at relatively low doses (∼300 and 1000 ng cm^2^) for 24 h did not trigger inflammatory cytokine production in alveolar epithelial cells co‐cultured with primary human monocyte‐derived macrophages and dendritic cells. In the only available human‐controlled inhalation study, it was shown that GO nanosheets of small and ultrasmall lateral dimensions at a concentration of 200 µg m^3^ for 2 h had no adverse effects on lung function and inflammatory markers [71]. Schlagenhauf et al. (2015) [23] investigated the toxicity of abraded MWCNT–epoxy nanocomposite (containing 1% MWCNTs) in the alveolar epithelial cell line (A549), showing no oxidative stress or cytotoxic and genotoxic effects after short‐term exposure (48 h) at concentrations up to 80 µg mL^−1^. Similarly, Carlin et al. (2024) [48] showed that abraded hexagonal boron nitride (*h*‐BN)‐TPU composites did not trigger acute (24 h) or sub‐acute (4 weeks of repeated exposure) toxicity in lung and skin cells. Bishop et al. (2017) [24] investigated the inhalation toxicity of carbon nanotubes (CNT)‐embedded polymer composites in mice, showing no influence on pulmonary injury or inflammation as compared to as‐produced CNT. In fact, they could show a significant reduction in toxicity after polymer coating.

## 3 Conclusions

The limited availability of safety studies addressing carbon‐based nanomaterial composites across their entire life cycle, integrating thorough material characterization of both pristine and released materials with comprehensive hazard assessment, has been recognized as an important knowledge gap [21]. This study addressed this gap by providing an integrated, life‐cycle‐oriented assessment of epoxy‐based graphene composites that combined in‐depth material characterization with an extensive inhalation hazard evaluation of released particles. The results demonstrated the successful detection and localization of few‐layer graphene within epoxy composites under conditions mimicking realistic production and end‐of‐life scenarios. Multiple complementary analytical techniques, including Raman spectroscopy, HR‐TEM, and ToF‐SIMS, were applied to assess the spatial distribution of graphene nanosheets within the product matrix and in abraded composite particles. HR‐TEM imaging revealed that abrasion predominantly generated composite fragments, with FLG remaining embedded in the epoxy matrix. No evidence of graphene protrusion or the release of free graphene nanosheets was observed. However, the presence of large FLG agglomerates in the epoxy matrix suggests that certain abraded FLG–epoxy fragments may contain relatively higher local concentrations of FLG. Such enriched fragments could result in increased FLG exposure during occupational handling or environmental release at the end‐of‐life stage.

The biological assessment revealed distinct responses to as‐produced few‐layer graphene compared with its epoxy‐based composites. While pristine FLG (prepared in 0.1% BSA‐water) did not impair alveolar macrophage viability, it induced a pronounced TNF‐α secretion, indicating pro‐inflammatory activity. In contrast, particles collected from abraded FLG‐epoxy composites induced only a limited TNF‐α response, suggesting that incorporation into the polymer matrix mitigated the inflammatory potential of FLG. The contribution of potential FLG oxidation to this response remains unclear and warrants further investigation. In bronchial and alveolar epithelial cell culture models, exposure to FLG, abraded epoxy, or abraded FLG‐epoxy composite particles did not affect cell viability. Sub‐toxic effects were observed only at relatively high exposure doses above 10 µg cm^2^, where abraded epoxy composites moderately reduced mucociliary clearance. These effects were not increased by the presence of FLG in composite material.

Overall, the data indicated a limited health risk associated with inhalation exposure to abraded epoxy and FLG‐epoxy composite particles during production and end‐of‐life scenarios, even at high exposure levels. By linking life‐cycle‐relevant material characterization with mechanistic toxicological endpoints, this study provides important insights for hazard assessment and supports the safe‐by‐design development of industrial graphene‐based composite materials. Finally, we would like to emphasise that this study evaluated one specific formulation of epoxy resin, hardener, and FLG. Since commercial products vary in composition, filler characteristics, manufacturing processes, and aging conditions, the findings may not fully reflect the broad spectrum of FLG‐containing products available on the market. Further studies using multiple formulations are recommended to assess the broader applicability of the presented methodology and findings.

## 4 Experimental Section

### 4.1 Synthesis, Dispersion and Characterization of Few‐layer Graphene

The graphene dispersion was prepared with the following method: in short, potassium graphite (KC8) was prepared by a standard procedure, that is, by reacting potassium with natural graphite at 150°C under an inert atmosphere. 20 g of KC8 were then dispersed in a liter of purified THF, and this mixture was stirred for six days with a magnetic stirrer (250 revolutions per minute (rpm). After stirring, the mixture was centrifuged at 2000 rpm for 20 min. The top two‐thirds of the solution were extracted with a pipette, oxidized with dry air, and mixed with water containing BSA (0.1%). THF was evaporated at room temperature to obtain a dispersion of graphene‐BSA used thereafter. For characterization, Raman measurements were performed using a Raman spectrometer (HORIBA Jobin Yvon Xplora). The Raman spectra were collected on the samples contained in a quartz cuvette placed on a holder fixed to a “‘macro’” (x4.5) objective of the Raman spectrometer at room temperature using the 1.58 eV laser excitation energies. The Raman acquisition was performed using a holographic grating with 1200 lines/mm and a CCD camera. The spectra were normalized to the T2g silicon peak at 521 cm^−^^1^ and the G band of Highly Ordered Pyrolytic Graphite (HOPG) at 1582 cm^−^^1^. The Raman spectrum of water was collected under the same conditions for the background. Micro‐Raman spectra were also collected on graphene films deposited on SiO2/Si substrates using a ×50 objective. UV–vis spectra of FLG dispersions were recorded between 200 nm and 800 nm (Jasco V670).

### 4.2 Fabrication of Epoxy/FLG Composites and Taber Abrasion

Epoxy (Epikote 827, Hexion) with the hardener (Baxxodur EC 301, Evonik) at a resin‐to‐hardener ratio of 100:33.52 (w/w) was used either in its neat form or supplemented with 1 wt.% few‐layer graphene (FLG, Carbon Waters). The epoxy resin was preheated at 60°C for 30–60 min to reduce viscosity and then mixed in a temperature‐controlled high‐shear mixer under a vacuum of ∼0.8 bar. The mixture was stirred up to 400 rpm for 3 min and then maintained at 150 rpm for another 3 min to ensure degassing. The resulting mixture was poured into sealed metal molds and cured first at 80°C for 12 h, followed by 120°C for 4 h. After cooling the plates to room temperature, the cured materials were demolded, cut to the required dimensions (23 × 21 × 0.4 cm), and used for mechanical abrasion. The abrasion was performed as described earlier [22]. Briefly, the composites were abraded using a Taber abrader (Model 5135, Taber, North Tonawanda, NY) equipped with S‐42 sandpaper strip wrapped around a CS‐0 wheel, with an applied weight of 1 kg, to simulate the sanding process on the composites' surfaces. Cyclic flow tests were performed for 5000 cycles at a frequency of 60 cycles·min^−^^1^ (one run corresponding to 84 min). The pump flow rate was maintained at 10.5 L min^−^^1^ throughout each run. The released particles were collected on a nucleopore polycarbonate filter (pore size: 0.2 µm, surface area: 40 mm^2^, Whatman, UK) via a vacuum inlet (flow rate 10 L min^−^^1^), connected to conductive silicon tubing (TSI, United States). For selected abrasion experiments with neat epoxy and FLG‐epoxy plates, particle size distributions were monitored in real time using a DMS500 Mk II Fast Particulate Analyzer (Cambustion, UK), which determines particle electrical mobility diameter. The instrument contains a cyclone pre‐classifier with a nominal cut‐off diameter of 1 µm; measurements were therefore restricted to the standard submicrometre size range of 5–1000 nm. Particle size distributions were exported as dN/dlogDp. A unimodal fitting routine was applied for neat epoxy measurements, whereas a bimodal fitting routine was used for FLG‐epoxy measurements to account for the possibility of multiple particle populations.

### 4.3 Dispersion of Abraded Epoxy and FLG‐Epoxy Composites and Initial Characterization Using DLS

For cell experiments, dispersion of abraded epoxy and FLG‐epoxy particles was prepared in endotoxin‐free BSA water (0.1%) by partly following the procedure suggested elsewhere [12]. Briefly, 2–5 mg of abraded powders were weighed in a sterile glass vial and supplemented with 0.1% of BSA‐water to achieve a 1 mg mL^−1^ stock suspension. The vial was vortexed for 1 min and then bath sonicated for 20 min first and then vortexed again for 1 min, followed by another 25 min, before use. The prepared dispersions were diluted accordingly in cell culture medium to achieve the desired exposure concentrations for experiments. The dispersed particles were then characterized for hydrodynamic size in water using the Zetasizer Nano ZS instrument (Malvern Instruments, UK).

### 4.4 Detection of FLG in Epoxy Composites

#### 4.4.1 Confocal Raman Microscopy (CRM)

Three‐dimensional Raman imaging of the FLG‐epoxy composite material was performed using a WITec alpha300 apyron (WITec GmbH, Ulm, Germany), equipped with a motorized, software‐controlled stage, a Zeiss LD EC Epiplan‐Neofluar HD DIC 50×/0.55 (Zeiss, Oberkochen, Germany) and a Newton DU970P‐BVF EMCCD camera (Oxford Instruments GmbH, Wiesbaden, Germany). Raman spectra were acquired using a 532 nm laser wavelength, an acquisition time of 0.5 s per spectrum and 1 mW laser power in a lateral resolution of 1.125 µm px^−1^ and a vertical resolution of 1 µm px^−1^. This was achieved using a 300 mm focal length spectrometer equipped with a 600 lines mm^−1^ grating blazed at 500 nm.

Data analysis of the measurement was conducted by the device‐specific analysis software Project 6.0 and the customized Raman database program TrueMatch 6.0 including reference spectra of both raw materials. 3D visualization was performed by a customized program using the software Wolfram Mathematica14.2.

#### 4.4.2 Secondary Ion Mass Spectrometry

All analyses were performed using an M6 Hybrid Secondary ion mass spectrometry (SIMS) instrument (IONTOF, Germany). Data evaluation was carried out with SurfaceLab 7.5 software (IONTOF, Germany). The instrument settings used for the data presented here are described below.

Depth profiling was carried out in duplicate using an Ar_1200_^+^​ gas cluster ion beam operated at 20 keV and 100 pA. The analyzed area was 300 × 300 µm^2^ with a raster size of 50 × 50 pixels. A total of 4 scans were acquired. Secondary ions were detected and mass‐separated using an Orbitrap analyzer. The helium pressure in the collision cell was set to 4.9 × 10^−2^ mbar. The injection time was 500 µs, operated in fast discard mode. Spectra were recorded over a mass range of *m/z* 70–1050. The mass resolution was set to R = 240 000, and the mass accuracy was better than 2 ppm for all evaluated signals.

The SIMS depth profiling measurements differed from the SIMS spectrometry settings only in the following aspects: a total of 420 scans were acquired, and depth profiles were recorded over a mass range of m/z 60–900.

SIMS imaging was performed using Bi_3_^+^​ primary ions operated at 30 keV and 0.2 pA. The field of view was set to 250 × 250 µm^2^ with a raster size of 512 × 512 pixels. Mass separation and detection were carried out using a time‐of‐flight (ToF) analyzer with a cycle time of 100 µs. All analyses were conducted in fast imaging mode at nominal mass resolution.

#### 4.4.3 High Resolution Transmission Electron Microscopy (HRTEM Analysis)

Epoxy blocks were cut for HRTEM by ultramicrotomy (Ultracut S; Leica Microsystems, Buffalo Grove, USA). Epoxy powders were embedded in another epoxy resin (Epofix, Struers GmbH, Willich, Germany) prior to ultramicrotomy. The sample was analyzed on a Thermo‐Fisher (Waltham, MA, USA) Talos F200i Transmission Electron Microscope in bright‐field mode at 200 kV. Images were taken with a Thermo‐Fisher Ceta 16 M camera. The magnification was calibrated using a Mag*i*cal (Technoorg Linda Ltd., Budapest, Hungary) calibration standard. Distances were either measured using the Thermo‐Fisher Velox 2 software package or by counting lattice fringes in the stacking‐direction of the FLG.

### 4.5 Alveolar Macrophage Assay (AMA)

#### 4.5.1 AMA Assay

NR8383 cells, a rat alveolar macrophage cell line (ATCC, USA No. CRL‐2192TM), were cultured in F‐12K medium (Merck KGaA, Germany) supplemented with 15% fetal bovine serum (FBS), 1% penicillin/streptomycin and 1% L‐glutamine (PAN biotech, Aidenbach, Germany).

For the macrophage assay, cells were seeded into 96‐well plates with 3 × 10^5^ cells /well in 5% FBS in F‐12K and kept at 37°C and 5% CO_2_ for 24 h. The next day the medium was replaced by serum‐free test material dispersions. Therefore, stock suspensions of test materials were prepared as described in Chapter 4.3. To achieve test concentrations, stock suspensions were mixed with an equal volume of double‐concentrated F‐12K medium, followed by serial dilution with F‐12K medium. After 16 h, cells were micrographed with an inverted phase contrast microscope (Zeiss Axiovert C40) equipped with an AxioCam II Camera to document the uptake of settled particles and cell morphology. Then, the supernatants were retrieved, centrifuged (200 xg, 10 min) and used to measure LDH (lactate dehydrogenase), GLU (glucuronidase) and TNF‐α.

LDH activity was determined using a Roche Cytotoxicity Kit (Merck KGaA, Germany) as described by the manufacturer; the resulting optical density (OD) was measured with an Infinite F200Pro plate reader (Tecan, Crailsheim, Germany). For GLU measurement, 50 µl of the supernatant was incubated with 50 µL 0.2 M sodium acetate buffer (pH 5) containing 13.3 mM p‐nitrophenyl‐D‐glucuronide and 0.1% Triton‐X‐100. The reaction was stopped after 90 min by adding 50 µL 0.2 M sodium hydroxide. All tests were performed in triplicates, corrected for cell‐free absorption and normalized to the results of the positive control (0.1% Triton X‐100 in cell culture medium).

TNF‐α was tested using an enzyme‐linked immunosorbent assay (ELISA) specific for rat TNF‐α (BioLegend, San Diego, USA). TNF‐α induction with 0.5 µg mL^−1^ lipopolysaccharide (LPS) served as a positive control in all assays. To exclude the influence of endotoxin possibly linked to FLG, a TNF‐α Expression Test (TET) was carried out. NR8383 cells were seeded in a 96‐well plate as described above and pre‐incubated for 30 min with 10 µM of the endotoxin inhibitor polymyxin B sulfate (Sigma–Aldrich), which was non‐cytotoxic for NR8383 cells (data not shown). The medium was then replaced by a mixture of polymyxin B (10 µM) and FLG (22.5‐180 µg mL^−1^) in serum‐free F‐12K medium. After 16 h, supernatants were analyzed for TNF‐α as described above.

#### 4.5.2 Correlative Confocal Raman and Scanning Electron Microscopy (SEM) of NR8383 cells:

NR8383 macrophages were seeded onto fibronectin‐coated coverslips (18 × 18 mm^2^) placed in wells of a 6‐well cell culture plate. To achieve a cell density suited for CRM, we seeded 2 × 10^6^ cells per well using 2 mL of F‐12K medium supplemented with 5% FCS and allowed cells to attach for 20 h. Then, particles (11.25 µg mL^−1^) were added in serum‐free F‐12K medium. After 16 h, cells were fixed with 2% glutaraldehyde and 1.8% paraformaldehyde dissolved in sodium phosphate buffer, pH 7.4, at room temperature for 10 min. Coverslips were washed extensively in phosphate buffered saline and dehydrated using a 7‐step series of increasing alcohol concentrations (30%, 50%, 70%, 80%, 90%, 96%, and 100%) for 10 min each. Then critical point drying was carried out, using a Leica EM CPD300 instrument. Samples were analyzed using correlative microscopy (CM), combining SEM (Phenom XL Desktop SEM, Thermo Fisher Scientific, Dreieich, Germany) and Raman microscopy (RM, Alpha300 apyron by WITec, Ulm, Germany). Both microscopes were equipped with fully motorized, software‐controlled stages to correlate a sample‐specific coordinate system to the stage's coordinate system. Raman microscopic imaging was performed using a Zeiss EC Epiplan‐Neofluar 100×/0.9 NA objective (Zeiss, Oberkochen, Germany). Video camera imaging resulted in an additional magnification factor of 11.6×. Raman spectra were acquired using a 532 nm laser wavelength, an acquisition time of 15 ms per spectrum and laser powers of 0.4, 1, or 5 mW (depending on the FLG content of the samples). This was achieved using a 300 mm focal length spectrometer equipped with a 600 lines mm^−1^ grating blazed at 550 nm and an EMCCD camera (Newton DU970P‐BVF, Oxford Instruments GmbH, Wiesbaden, Germany). Three‐dimensional Raman imaging of at least five single cells per sample was performed by acquiring 100 spectra per µm^2^ and stacks with a vertical resolution of 1 µm. For each sample, 50 brightfield images were acquired at random positions with a pixel resolution of 113.3 nm. The samples were prepared for SEM imaging by sputter coating with a 10 nm thick gold layer using a Leica EM ACE600 Sputter Coater. The exact same locations as in brightfield imaging were imaged using secondary electron (SE) imaging at an acceleration voltage of 5 kV and a pixel resolution of 124.5 nm, which corresponds to a magnification of 500×. Additional SEM images of 3D‐measured single cells were acquired at a magnification of 1500×, with a pixel resolution of 41.3 nm. High‐resolution SEM imaging and tilted image acquisition was performed with a Hitachi SU8230 SEM (Hitachi, Tokyo, Japan) in different magnifications. Data analysis of the three‐dimensional Raman measurements was conducted by the device‐specific analysis software Project7.0 and the customized Raman database program TrueMatch7.0, including reference spectra of the raw materials. 3D visualization was performed by using a customized script in Wolfram Mathematica 14.3.

### 4.6 Primary Human Bronchial (MucilAirTM) and Alveolar (AlveolAirTM) Epithelial Cell Cultures

#### 4.6.1 MucilAir and AlveolAir Cultures

MucilAir is a fully differentiated bronchial cell model (EP01MD, Epithelix) reconstituted using primary human bronchial epithelial cells collected from five healthy donors and pooled. Primary cells are obtained from human biological samples collected under ethical approval and with informed donor consent. Each tissue batch is accompanied by a certificate of analysis detailing donor information and quality control data. The cells were seeded on a microporous membrane of Transwell inserts (24‐well format, Oxyphen, Lottstetten, Germany) and cultured in MucilAir culture medium (EP04MM, Epithelix). After reaching confluency, the cell cultures were turned into an ALI condition and maintained in a CO_2_ incubator (5% CO_2_, 100% humidity, 37°C). The differentiated epithelium contains ciliated, goblet, and basal cells and exhibits the presence of a well‐developed transepithelial barrier, conductive cilia, mucus production, and clearance.

AlveolAir is a fully primary human alveolar model reconstructed from lung parenchyma of healthy donors. The lung biopsies were obtained from the tissue collection center. The epithelial and endothelial cells were isolated from distal lungs by enzymatic digestion and expanded in cell culture medium. The cells were seeded on the microporous membrane of Transwell inserts (24‐well format, Oxyphen, Lottstetten, Germany) and grown in a AlveolAir culture medium (Epithelix, EP74AL). After cell cultures achieved confluency, they were brought into an ALI for one week and then differentiated. Fully differentiated cell cultures sustained the presence of type I alveolar pneumocytes (CAV‐1 and PDPN‐positive cells) and type II pneumocytes (HTII‐280‐positive cells and lamellar bodies), formed tight junctions on the apical side of the Transwell insert. The AlveolAir model exhibits a relevant barrier function over 30 days (trans‐epithelial electrical resistance, TEER) and secretes surfactant protein C [72].

Both MucilAir and AlveolAir cell culture experiments were conducted according to the “Declaration of Helsinki on biomedical research (Hong Kong amendment, 2013)” and received approval from the local ethics commission. The donors of the lungs, or their relatives, have given, read and signed an informed consent form.

Repetitive exposure to materials was performed by adding 10 µL (1, 10, 25, and 50 µg cm^2^) of FLG, abraded epoxy and abraded FLG‐epoxy suspensions to the apical compartment of cell cultures once a day for four consecutive days. Following exposure, the cell cultures and basolateral conditioned medium were collected for different cytotoxicity and immunomodulatory assays as described below.

#### 4.6.2 LDH Release

LDH release (indicative of cell membrane rupture) was used for cytotoxicity assessment of FLG and abraded composite materials in both MucilAir and AlveolAir cell culture models. Conditioned medium from cells exposed to Triton X‐100 (10%) for 24 h was used as a positive control. The assay was conducted using Cytotoxicity LDH Assay Kit‐WST (Dojindo, CK 12–20, TU797, Japan) by following the manufacturer's instructions. Briefly, 50 µL of basolateral conditioned medium was added to a 96‐well plate and mixed with 50 µL LDH substrate, followed by incubation at room temperature for 30 min. Finally, the absorbance of wells containing samples was measured at 490 nm using a microplate reader (Victor Nivo Microplate Reader, PerkinElmer Inc., USA). Absorbance was corrected with a blank control (cell culture medium only) and results were plotted as a percentage of viable cells compared to the positive control. The experiments were carried out with three biological replicates.

#### 4.6.3 Transepithelial Electrical Resistance

Transepithelial electrical resistance (TEER) measurements of cell cultures were performed every day up to 4 days using an STX2 electrode with an EVOMX volt‐ohmmeter (World Precision Instruments UK, Stevenage). Briefly, 200 µL and 700 µL of 0.9% saline solution were added to the apical and basolateral compartments of the cell cultures, respectively. Resistance values (Ω) measured in each cell culture, when was then transformed to TEER values (Ω.cm^2^) by applying formula: TEER (Ω·cm^2^) = (resistance value (Ω) – 100(Ω)) × 0.33 (cm^2^), where 100 Ω is the resistance of the membrane and 0.33 cm^2^ is the total surface of the epithelium in the Transwell inserts.

#### 4.6.4 Cilia Beating Frequency

MucilAir cell cultures retain conducting cilia that perform the mucociliary clearance function. To understand whether exposure to FLG and abraded composite particles affects Cilia beating frequency (CBF), we recorded the frequency (Hz) of beating cilia in cell cultures using a Sony XCD V60 camera coupled to an Olympus BX51 microscope and a PCI card. For analysis, a total of 256 images were captured at a frequency of 125 frames per second at room temperature and CBF was analyzed using CiliaX software (Epithelix).

#### 4.6.5 Mucociliary Clearance

The mucociliary clearance (MCC) function of MucilAir cell cultures was determined by following the procedure described elsewhere [51]. Briefly, polystyrene microspheres (30 µm diameter, Sigma–Aldrich, cat no. 84135) were applied to the apical surface of cell cultures. and movement of microbeads was recorded using a Sony XCD‐U100CR camera connected to an Olympus BX51 microscope. The average velocity of bead movement (µm/s) from three independent cultures was calculated using the ImageProPlus 6.0 software.

#### 4.6.6 Interleukin (IL)‐8 Release

The IL‐8 release from MucilAir and AlveolAir cell culture in basolateral conditioned medium was determined after day 2 and day 4 of exposure using an ELISA kit (BD Biosciences 555244). Briefly, all the samples collected from the repeated exposure experiment were diluted to 1:1000 times in the assay diluent buffer provided in the ELISA kit and processed further according to the manufacturer's instructions. In the end, the absorbance was recorded at 450 nm using a multiplate ELISA reader (Victor Nivo Microplate Reader, PerkinElmer Inc., USA). The results were converted into ng/mL using values from the standard curve and then multiplied by the dilution factor.

#### 4.6.7 Immunocytochemistry

MucilAir cell cultures collected after 4 days of repetitive exposures to 0.5% BSA (vehicle control), FLG and abraded epoxy and FLG‐epoxy were washed thrice using 1x PBS (Gibco), followed by fixation using Histofix (4% formaldehyde, Sigma–Aldrich, Germany) for 20 min. Next, the cultures were permeabilized using 0.2% Triton X‐100 and blocked using goat serum (10%, Sigma–Aldrich, Germany) at room temperature for 1 h. The cell cultures were then stained overnight at 4°C in primary goat anti‐ZO‐1 (1:200, PA5‐19090, Invitrogen, Germany) and rat anti‐tubulin (1:200, ab6161, Abcam, Germany) antibodies. The next day, the cultures were washed thrice with 1 × PBS and then stained with Alexa Fluor 488‐conjugated goat anti‐mouse (1:400, Invitrogen, Switzerland) and Alexa Fluor 555‐conjugated goat anti‐rat (1:400, Invitrogen, Switzerland) secondary antibodies for 1 h at RT. Finally, the cultures were stained with DAPI (1:1000, Invitrogen) for 10 min, washed and mounted on a glass slide using Mowiol488 (Sigma–Aldrich, Germany). The cells were visualized using CLSM (LSM 780, Zeiss, Germany) at 63x magnification. The images were processed using Zen software (version 2.6).

#### 4.6.8 Scanning Electron Microscopy

The interaction of materials with the cellular surface of MucilAir cultures and the resulting cellular morphology were assessed with SEM following the SEM sample processing protocol from Wang et al. (2025) [51], with the exception that a 10 nm Au/Pd layer was sputter‐coated instead of 10 nm carbon before the cellular surfaces were imaged at different magnifications with an Axia ChemiSEM (Thermo Fisher Scientific) microscope at 10 kV and 65 pA using an ETD detector.

#### 4.6.9 HRTEM

For the detection of material uptake, AlveolAir cells on inserts were processed for HRTEM following our TEM sample processing protocol [51], including fixation, osmium staining, dehydration, a graded Epon series and embedding in molds. Ultrathin sections (80‐100 nm) were then prepared using an ultramicrotome (Ultracut S; Leica Microsystems, Buffalo Grove, USA) and placed on formvar‐coated copper grids. The sample was analyzed on a Thermo‐Fisher (Waltham, MA, USA) Talos F200i Transmission Electron Microscope in bright‐field mode at 200 kV. Images were taken with a Thermo‐Fisher Ceta 16 M camera.

#### 4.6.10 48‐Plex Cytokine‐Chemokine Array

Cytokine and chemokine release in the basolateral medium of primary human bronchial and alveolar cell cultures was quantified after 4‐day exposure to FLG, Epoxy composite, or FLG‐Epoxy composite by Luminex based Human Cytokine/Chemokine Panel A 48‐Plex Discovery Assay Array (HD48A, Eve Technologies Corp., Canada), which included EGF, Eotaxin, FGF‐2, FLT‐3L, Fractalkine, G‐CSF, GM‐CSF, GROα, IFNα2, IFNγ, IL‐1α, IL‐1β, IL‐1RA, IL‐2, IL‐3, IL‐4, IL‐5, IL‐6, IL‐7, IL‐8, IL‐9, IL‐10, IL‐12p40, IL‐12p70, IL‐13, IL‐15, IL‐17A, IL‐17E/IL‐25, IL‐17F, IL‐18, IL‐22, IL‐27, IP‐10, M‐CSF, MCP‐1, MCP‐3, MDC, MIG/CXCL9, MIP‐1α, MIP‐1β, PDGF‐AA, PDGF‐AB/BB, RANTES, sCD40L, TGFα, TNFα, TNFβ, and VEGF‐A. Basolateral medium was collected, centrifuged at 500 g for 20 min at 4°C, and filtered through a 0.22 µm micro‐column to remove residual cellular debris. The conditioned medium from cells exposed to CytoMix (consisting of 0.5 µg mL^−1^ of TNF‐α (Biolegend, Ref 570104), 200 µg mL^−1^ of LPS (Sigma, Ref L9143), and 1% fetal calf serum (Bioconcept, Ref 2‐01F16‐I)), was applied as the positive control. The experiments were performed with three biological replicates. Statistical significance was determined using an unpaired two‐tailed t‐test. For data visualization, Z‐scored row entries were plotted as a heat map using the pheatmap library (version 1.0.12, Kolde, R. pheatmap: Pretty Heatmaps, 2022).

### 4.7 ALIsens Cell Culture

#### 4.7.1 Cell Lines and ALIsens Coculture Workflow

The human cell lines A549, THP‐1 and EA.hy926 were obtained from ATCC. A549 cells were cultured in DMEM supplemented with 10% (v/v) FBS, THP‐1 cells were maintained in RPMI‐1640 containing 10% (v/v) FBS, 25 mM HEPES, and 50 µM β‐mercaptoethanol and EA.hy926 cells were grown in DMEM supplemented with 10% (v/v) FBS and 25 mM HEPES. Cells were maintained in a humidified atmosphere with 5% CO_2_ at 37°C and tested regularly for mycoplasma contamination (MycoAlert Mycoplasma detection kit, Lonza). The correct identity of each cell line was confirmed via Human STR profiling cell authentication service provided by American Type Culture Collection (ATCC, Manassas, VA, USA).

The ALIsens model was seeded as described in Chary et al. (2019) [63]. On day 0, THP‐1 cells differentiation into macrophage‐like cells was started by seeding 4 × 10^5^ cells mL^−1^ in complete THP‐1 medium with addition of phorbol‐12‐myristate‐13‐acetate (PMA; 20 ng mL^−1^; Merck). On day 2, differentiation of PMA‐treated cells was enhanced by removing the PMA‐containing medium and replacing it with fresh THP‐1 complete medium for an additional 4 days.

On day 2, EA.hy926 and A549 cells were seeded on CellQART cell culture inserts (surface area of 4.5 cm^2^; 5 µm pore size; high pore density PET membranes for 6‐well plates; Sabeu, Northeim, Germany). EA.hy926 cells were seeded on inverted CellQART inserts (2.4 × 10^4^ cells/cm^2^). After attachment to the basolateral side, the inserts were returned to their upright position before A549 cells were seeded on the apical side (6 × 10^4^ cells cm^2^). On day 5, medium was replaced by complete coculture medium (medium composed of 75% DMEM, 15% RPMI‐1640, and 10% IMDM) containing 10% of FBS. On day 6, differentiated THP‐1 (2.4 × 10^4^ cells cm^2^) cells were added to the apical compartment on top of the confluent A549 layer, and undifferentiated THP‐1 cells (1 × 10^6^ cells mL^−1^) were added to the basolateral compartment in coculture medium containing 1% FBS. After 4 h, the medium from the apical compartment was removed and the coculture was cultivated overnight at the ALI until exposure.

On day 7, cells were exposed to FLG‐EPOXY (1, 10, 50 and 100 µg cm^2^), Pristine FLG (1, 5, 10 and 30 ug cm^2^) and abraded EPOXY (1, 10, 50 and 100 µg cm^2^). A stock solution of each particle was made using 0.1% BSA in endotoxin‐free ultrapure water. A stock solution of each particle type was prepared in 0.1% BSA in endotoxin‐free ultrapure water. Working dilutions were then prepared in 1.65 mL coculture medium containing 1% FBS to reach the desired µg/cm^2^ concentrations. Cells were also exposed to vehicle control consisting of coculture medium with 1% FBS and stock of BSA in ultrapure water. A positive control for the flow cytometry measurements was also performed by exposing the cells to a stimulation mix comprising 10 µg mL^−1^ of LPS and 20 ng mL^−1^ of TSLP.

On day 8, 24 h after the exposures, Alamar Blue assay and flow cytometry measurements were performed. Supernatant was collected, centrifuged at 1000 g and stored at −80°C for cytokines measurements by Luminex.

#### 4.7.2 Assessment of Cell Viability

24 h after exposure, the cell viability of the cocultures was assessed using 400 µM of Alamar Blue diluted in culture medium. Briefly, cell culture inserts were transferred to a new 6‐well plate and 400 µM of Alamar Blue diluted in coculture medium was added in both compartments. 20 uL of Alamar Blue stock solution at 20 mM was added to the THP‐1 cells in suspension in the old plate. After 1 h incubation at 37°C in the dark, medium from each compartment (apical, basolateral and THP‐1 cells) was collected and the fluorescence measured at 530 nm excitation and 590 nm emission using a fluorescence microplate reader (Spark 20 M, Tecan, Mechelen, Belgium). Filters used were 530/25 (excitation) and 590/20 (emission), respectively. Relative viability was determined by using the following calculation:

$$relative\;viability\left(\%\right)=\frac{fluorscence\;sample-blank}{fluorescence\;vehicle\;control-blank}$$

#### 4.7.3 Flow Cytometry Measurements

After 24 h exposure, THP‐1 cells were collected from the basolateral compartment of the wells and were stained with Phycoerythrin (PE) ‐, allophycocyanin (APC)—or Brilliant Blue 515 (BB515)—labelled monoclonal antibodies and isotype‐matched controls (mouse IgG1). PE anti‐CD86 (clone 2331 [FUN‐1]), BB515 anti‐CD54 (clone HA58) and APC‐anti TSLPr (clone 1F11) were purchased from BD Pharmingen (Heidelberg, Germany).

Surface marker expression was measured using FACS Celesta cell analyzer and analyzed with the FlowJo software V10. Sytox Blue‐positive cells (dead cells) were excluded from the analysis. Relative mean fluorescence intensities (rMFI) were expressed in % and calculated as follows:

$$rMFI\left(\%\right)\frac{MFI\;of\;antibodies\;stained\;sample-MFI\;of\;isotypes\;stained\;sample}{MFI\;of\;antibodies\;stained\;vehicle\;control-MFI\;of\;isotypes\;stained\;vehicle\;control}\times 100$$

#### 4.7.4 Cytokine‐Chemokine Screening

Cytokines release (VEGF, IL‐6, IL‐7, IL‐1Rα,, IL‐10, IL‐12, IL‐12p40, IL‐8, eotaxin, MIP‐1α, GRO‐α, TNF‐α, MCP‐3, MIP‐1β, IFN‐γ, RANTES, GCSF, GM‐CSF, and MCP‐1) in both the apical and basolateral compartments was quantified after 24 h of exposure to 10 µg cm^2^ of FLG, Epoxy composite and FLG‐Epoxy composite as well as to the stimulation mix, using a Bio‐Plex 3D system with the Bio‐Plex Pro Human Cytokine Screening Panel (Biorad) according to manufacturer's instructions. Apical and basolateral media were collected, centrifuged at 1000 g for 10 min at 4°C and stored at −80°C until analysis.

### 4.8 Statistical Analysis

Statistical analysis was performed and presented with GraphPad Prism 10 (GraphPad Software, USA). Details of the statistical methods used for each specific data set are described in the corresponding figure legends.

## Author Contributions

**Govind Gupta**: conceptualization, investigation, formal analysis, writing – original draft, writing – review and editing, visualization, supervision, project administration. **Jimmy Vernaz**: investigation, writing – review and editing. **Antje Vennemann**: investigation, formal analysis, visualization, writing – review and editing. **Maike Stange**: investigation, formal analysis, visualization, writing – review and editing. **Asmus Meyer‐plath**: investigation, formal analysis, visualization. **Servane Contal**: investigation. **Sarah Zehnder**: investigation. **Ziting Wang**: investigation, formal analysis, visualization. **Aline Chary**: investigation, formal analysis, visualization, project administration, writing – review and editing. **Stephanie Eitner**: investigation. **Philipp Mueller**: investigation, writing – review and editing, visualization. **Vera M. Kissling**: investigation, writing – review and editing, supervision. **Samuel Constant**: project administration, funding acquisition, writing – review and editing. **Lucie Chupin**: formal analysis, writing – review and editing. **Daniel Breitenstein**: investigation, formal analysis, writing – review and editing. **Birgit Hagenhoff**: writing – review and editing. **Tommaso Serchi**: supervision, funding acquisition, writing – review and editing. **Bernadett Boda**: investigation, formal analysis, project administration, writing – review and editing. **Martin Wiemann**: conceptualization, writing – review and editing, visualization, supervision, project administration, funding acquisition. **Peter Wick**: conceptualization, writing – review and editing, supervision, project administration, funding acquisition. **Tina Buerki‐thurnherr**: conceptualization, writing – review and editing, visualization, supervision, project administration, funding acquisition.

## Conflicts of Interest

The authors declare no conflicts of interest.

## Supporting information




**Supporting File 1**: smll76506‐sup‐0001‐SuppMat.docx.

**Supporting File 2**: smll76506‐sup‐0002‐VideoS1.mp4.

**Supporting File 3**: smll76506‐sup‐0003‐VideoS2.mp4.

**Supporting File 4**: smll76506‐sup‐0004‐VideoS3.mp4.

**Supporting File 5**: smll76506‐sup‐0005‐VideoS4.mp4.

## Data Availability

The data that support the findings of this study are available from the corresponding author upon reasonable request.
