# Supplementary material for: Detection and Inhalation Hazard Assessment of Wear Particles Arising From Abrasion of Market‐relevant Graphene Reinforced Composites
Source: Small. 2026 Oct 30;22(63):e76506. doi: 10.1002/smll.76506 (PMC13755991; doi:10.1002/smll.76506)
Supplement: Supplementary file 1 [file SMLL-22-e76506-s003.docx]

Supporting Information

**Detection and inhalation hazard assessment of wear particles released by mechanical abrasion of market-relevant graphene composites**

Govind Gupta^1^, Jimmy Vernaz^2^, Antje Vennemann^3^, Maike Stange^4^, Asmus Meyer-Plath^4^, Servane Contal^5^, Sarah Zehnder^1^, Ziting Wang^1^, Aline Chary^5^, Philipp Mueller^6^, Stephanie Eitner^1^, Vera M. Kissling^1^, Samuel Constant^2^, Lucie Chupin^7^, Daniel Breitenstein^8^, Birgit Hagenhoff^8^, Tommaso Serchi^5^, Bernadett Boda^2^, Martin Wiemann^3^, Peter Wick^1^, Tina Buerki-Thurnherr^1*^

*^1^Nanomaterials in Health Laboratory, Swiss Federal Laboratories for Materials Science and Technology (Empa), St. Gallen, Switzerland; ^2^Epithelix Sàrl, Geneva, Switzerland; ^3^IBE R&D Institute for Lung Health GmbH, Münster, Germany; ^4^Federal Institute for Occupational Safety and Health (BAuA), Materials and Particulate Hazardous Substances, Berlin, Germany; ^5^Luxembourg Institute of Science and Technology (LIST), 41 Rue du Brill, L-4422 Belvaux, Luxembourg; ^6^BASF SE, Solid-State Microstructures, Carl-Bosch-Strasse 38, 67056 Ludwigshafen am Rhein, Germany; ^7^Carbon Waters, Pessac, France; ^8^Tascon GmbH, Münster, Germany*

**Corresponding Author*

*Dr. Tina Buerki-Thurnherr*

*tina.buerki@empa.ch*

*ORCID ID: 000-0003-3723-6562*

**Scheme S1:** Fabrication of epoxy and FLG-epoxy plates, and mechanical abrasion to collect respective composites mimicking end-of-life scenario. 300 g of Epoxy (Epikote 827), 100.56 g of hardener (Baxxodur EC 301) with or without 1% FLG (1%) were mixed, and plates of fixed dimensions (23×21×0.4 cm) were prepared by filling the mixture in molds and curing them for two consecutive cycles of 12 h and 4 h at 80 °C and 120 °C, respectively. The cured plates were collected and used for mechanical abrasion using a Taber abrader. The abrasion was performed at a fixed speed of the wheel running 60 cycles/min and placed in a fume hood. The abraded particles passed through a metallic probe (flow rate 10.5 L/min) and collected on polycarbonate membrane filters (pore size: 0.22 μm, diameter: 47 mm). After each run (5000 cycles 84 min), the filters were taken off and abraded composite particles were collected in a sterile Falcon tube by scraping the membrane using a sterile spatula.

**Figure S1:** Orbi-SIMS spectrum of FLG and epoxy composites before and after polymerization and mechanical abrasion. (a-b) Spectrum of epoxy composite (a) and FLG-epoxy composites (b). The observed peaks indicate the presence of epoxy fragments (C_14_H_11_O_2_^-^; C_18_H_19_O_3_^-^), laurylsulfate (C_12_H_25_SO_4_^-^, only in FLG-epoxy plate surface), alkylbenzene sulfonate (C_18_H_29_SO_3_) and palmitate (C_16_H_31_O_2_^-^). A.u.: arbitrary units.

**Figure S2:** Depth profile of FLG containing epoxy composite plates showing the presence of the indicated surfactant compound and other potential contaminants in the outermost surface.

**Figure S3:** Electrical mobility diameter distributions measured with a DMS500 during abrasion of neat epoxy and FLG-epoxy plates. Distributions are shown as dN/dlogDp as provided by the instrument software. Measurements were restricted to the submicrometre size range of the instrument.

**Figure S4:** Confocal laser scanning microscopy (CLSM) images of pHBE cell cultures showing ciliary distribution (red), tight junction (green), and nucleus (blue).

**Figure S5:** SEM images showing ciliary interactions of FLG, Epoxy composite and FLG-Epoxy compsite (blue arrows) as well as clearly observable mucus accumulation (yellow arrows) and no significant ciliary morphology changes on treated pHBE cell cultures after 4 days of repeated exposure in contrast to untreated control cells.

**Figure S6:** Cytotoxicity and inflammatory response of primary bronchial epithelial cell cultures (MucilAir^TM^) after 2 and 4 days of repetitive exposure to FLG and epoxy composites. (a-c) Lactate dehydrogenase (LDH, indicating cell membrane rupture) release from pHBE cell cultures after exposure to (a) FLG, (b) Epoxy composite and (c) FLG-Epoxy composite. (d-f) Interleukin (IL)-8 release (indicating inflammatory response) from pHBE cell cultures after exposure to (d) FLG, (e) Epoxy composite and (f) FLG-Epoxy composite. Positive controls: 10% Triton X-100 or to CytoMix™. Data are presented as mean ± SD (n=3). Each cell culture was reconstituted from primary cells pooled from five different donors (n=5). Statistical significance was calculated between vehicle control (VC) and individual treatments by applying two-way ANOVA with Dunnett’s multiple comparisons test. ***p<0.001

**Figure S7:** Cytokine-chemokine responses of pHBE cell cultures after 4 days of repetitive exposure to epoxy and FLG composites or FLG alone at 10 µg/cm^2^. Data presented in the heat map were clustered using mean values (n=3) of detected cytokines and chemokines. Statistical significance was calculated between individual treatments *versus* vehicle control by applying the T-test. The p-value < 0.05 was considered statistically significant. *p < 0.05, **p < 0.01, ***p < 0.001, ****p < 0.0001 indicates statistically significant with respect to the vehicle control (denoted as VC).

**Figure S8:** TNF-α expression test (TET) to exclude an endotoxin contamination of the FLG suspension. The graph shows that polymyxin-B (10 µM) was unable to block FLG-mediated TNF-α release, confirming the strong pro-inflammatory potential of FLG nanosheets in NR8383 cells. Dashed lines indicate the efficacy of polymyxin B, which reduced the TNF-α production of cells induced with a high concentration of LPS (0.5 µg/mL).

**Figure S9:** Cellular uptake of abraded epoxy (a) and FLG-epoxy (b) particles in primary alveolar epithelial cell cultures after day 4 of exposure. TEM micrographs demonstrate uptake of several particles (white arrow; a', b') and HRTEM confirmed the presence of internalized FLG in abraded FLG-epoxy particles (b''-b''').

**Figure S10:** Cytotoxicity of FLG and epoxy composites on primary alveolar epithelial cell cultures. (a-c) LDH release from cell cultures after day 2 and day 4 of exposure to FLG alone (a), epoxy composite (b), and FLG-epoxy composite (c). Data are presented as mean ± SD (n=3). Statistical significance was calculated between vehicle control (VC) and individual treatments by applying two-way ANOVA with Dunnett’s multiple comparisons test. ***p<0.001

**Figure S11:** Cytokine-chemokine responses of primary alveolar cell cultures after 4 days of repetitive exposure to FLG alone or epoxy and FLG-epoxy composites at 10 µg/cm^2^. Data are presented as mean ± SD (n=3). Statistical significance was calculated between individual treatments *versus* vehicle control by applying the T-test. The p-value < 0.05 was considered statistically significant.
